# The effectiveness and safety of proton beam radiation therapy in children and young adults with Central Nervous System (CNS) tumours: a systematic review

**DOI:** 10.1007/s11060-023-04510-4

**Published:** 2024-01-31

**Authors:** Jayne S. Wilson, Caroline Main, Nicky Thorp, Roger E. Taylor, Saimma Majothi, Pamela R. Kearns, Martin English, Madhumita Dandapani, Robert Phillips, Keith Wheatley, Barry Pizer

**Affiliations:** 1 Cancer Research UK Clinical Trials Unit (CRCTU), Institute of Cancer and Genomic Sciences, University of Birmingham, Birmingham, UK; 2 The Clatterbridge Cancer Centre, Liverpool, UK; 3 The Christie Hospital Foundation Trust Proton Beam Therapy Centre, Manchester, UK; 4 College of Medicine, Swansea University, Swansea, UK; 5 Birmingham Women’s and Children’s Hospital NHS Foundation Trust, Birmingham, UK; 6 National Institute for Health Research (NIHR) Birmingham Biomedical Research Centre, University Hospitals Birmingham NHS Foundation Trust, Birmingham, UK; 7 Children’s Brain Tumour Research Centre, University of Nottingham, Nottingham, UK; 8 Queen’s Medical Centre, Nottingham University Hospitals’ NHS Trust, Nottingham, UK; 9 Centre for Reviews and Dissemination (CRD), University of York, York, UK; 10 Alder Hey Children’s NHS Foundation Trust, Liverpool, UK; 11 University of Liverpool, Liverpool, UK

**Keywords:** Children, CNS tumours, Proton beam radiotherapy, Brain, Photon beam, Systematic review

## Abstract

**Background:**

Central nervous system (CNS) tumours account for around 25% of childhood neoplasms. With multi-modal therapy, 5-year survival is at around 75% in the UK. Conventional photon radiotherapy has made significant contributions to survival, but can be associated with long-term side effects. Proton beam radiotherapy (PBT) reduces the volume of irradiated tissue outside the tumour target volume which may potentially reduce toxicity. Our aim was to assess the effectiveness and safety of PBT and make recommendations for future research for this evolving treatment.

**Methods:**

A systematic review assessing the effects of PBT for treating CNS tumours in children/young adults was undertaken using methods recommended by Cochrane and reported using PRISMA guidelines. Any study design was included where clinical and toxicity outcomes were reported. Searches were to May 2021, with a narrative synthesis employed.

**Results:**

Thirty-one case series studies involving 1731 patients from 10 PBT centres were included. Eleven studies involved children with medulloblastoma / primitive neuroectodermal tumours (n = 712), five ependymoma (n = 398), four atypical teratoid/rhabdoid tumour (n = 72), six craniopharyngioma (n = 272), three low-grade gliomas (n = 233), one germ cell tumours (n = 22) and one pineoblastoma (n = 22). Clinical outcomes were the most frequently reported with overall survival values ranging from 100 to 28% depending on the tumour type. Endocrine outcomes were the most frequently reported toxicity outcomes with quality of life the least reported.

**Conclusions:**

This review highlights areas of uncertainty in this research area. A well-defined, well-funded research agenda is needed to best maximise the potential of PBT.

Systematic review registration.

PROSPERO-CRD42016036802.

**Supplementary Information:**

The online version contains supplementary material available at 10.1007/s11060-023-04510-4.

## Introduction

Central Nervous System (CNS) tumours account for approximately 25% of all childhood neoplasms. Improvements in multimodality treatment regimens including surgical resection, focal and craniospinal radiotherapy (RT) and chemotherapy, have led to the 5-year overall survival rate of around 75% for this group of tumours in UK children [1]. Conventional RT (photon RT), which uses photon (x-ray) beams to target cancer cells, has made a significant contribution to survival, however it is associated with long-term adverse effects resulting from damage to adjacent healthy tissue which can lead to long-term cognitive, developmental and behavioural dysfunction [2–4]. These are caused by a combination of the direct and indirect impact of the tumour itself and also patient and treatment related parameters. There has been increasing interest in the potential of proton beam therapy (PBT) to reduce these late adverse events. Compared to photon RT, PBT is associated with smaller volumes of non-target irradiated normal tissue [5–9] largely due to the near complete elimination of exit dose [10]. Based on modelling assumptions from dosimetric studies, PBT has been adopted as the primary RT treatment modality for selected paediatric CNS tumours in several healthcare systems worldwide. In turn it is assumed that the radiodosimetric advantage of PBT will translate into improved clinical benefits such as a reduction in neuro-psychological sequalae and a lower incidence of radiotherapy induced second tumours.

The utility of systematic reviews to summarise research evidence in a non-biased, reproducible and transparent way is well established. Our initial scoping review identified three published systematic reviews that had investigated the effectiveness of PBT [11–13]. In all three, searches were up to 2014, meaning they were all out of date. In addition one had missing studies [11], one included both adults and children with brain tumours [12] and one included all paediatric cancers, not just brain tumours [13]. With the recent opening of two UK NHS proton facilities in Manchester at The Christie Hospital and in London at the University College London Hospital (UCLH) [14] [15], it is timely for an up-to-date assessment of the evidence base.

The aim of this systematic review was to evaluate the effectiveness of PBT in children and young adults with CNS tumours to assess the potential benefits and harms and identify any research gaps.

## Methods

### Protocol

Standard systematic review methodology aimed at minimising bias as recommended by the Cochrane Collaboration was employed and reported in accordance with Preferred Reporting Items for Systematic Reviews and Meta-Analysis (PRISMA) guidelines [16]. For more details see the published protocol (PROSPERO CRD42016036802) [17].

### Eligibility criteria

Studies were included in the review if they met the following criteria:

#### Population

Children and young adults (age up to 25 years) with any type of CNS tumour. Studies had to have a minimum sample size of nine patients [18, 19]. Studies with a mix of older adults and children/young adults were included provided that patient baseline data and outcomes were reported separately for children/young adults. Studies reporting a mix of tumour types were initially included, however, it was felt that disease-specific data within these was at risk of reporting bias, therefore a decision to exclude them was made at data extraction where this was suspected.

#### Intervention

PBT, used alone or as part of a multimodality treatment regimen.

#### Comparator

For comparative studies, we accepted conventional photon external beam radiation including three-dimensional (3D) conformal techniques or intensity-modulated radiation therapy (IMRT) including arc therapy, stereotactic radiosurgery, or brachytherapy used alone or as part of a multimodality treatment programme.

#### Study designs/publication type

Published full text studies that were either randomised controlled trials (RCTs), non-randomised controlled studies, phase II single arm trials and case series studies were included.

### Search strategy

Searches were undertaken from database inception to May 2021 in twelve bibliographic databases including MEDLINE, EMBASE and the Cochrane Library (search strategy provided in Supplementary Information (SI 1 and SI 2)). No language, publication or study design filters were applied. Reference lists of relevant studies were reference checked and clinical experts in the field consulted.

### Study selection

Study selection was undertaken independently by multiple reviewers in the author group and disagreements resolved by discussion, with JSW and BP making the final decisions.

### Data items and extraction process

Data extraction and risk of bias assessment were undertaken by one reviewer and checked by a second. Data was collected on specially designed pro-forma in Word and included data on patient characteristics, treatment regimens, and outcome measures. Proton radiation dose was measured in SI units of Gray Relative Biological Effectiveness (Gy_RBE_). Missing data was not imputed (SI 3). Risk of bias was assessed using a checklist designed to assess the validity of case series [17, 20], covering the domains of selection, detection and attrition bias. Additional criteria to assess the adequacy of the sample size, methods of analysis, outcome reporting and external validity of the study were also added and reported as a global assessment of the data set—see questions 13–17 of the data extraction sheet (SI 3).

### Effect measures

Effect measures were categorised as tumour related or toxicity related. Tumour related included: overall survival (OS), progression-free survival (PFS), event-free survival (EFS), recurrence-free survival (RFS), local and distant failure rates (LFR/DFR), response rates (RR), nodular failure-free survival (NFFS), and cystic failure-free survival (CFFS). Toxicity-related included: short- and long-term adverse events, such as necrosis, endocrine insufficiencies, ototoxicity and health related quality of life (HRQoL).

### Synthesis methods

Results were grouped according tumour type, and reported in a standard format across the tumour types, allowing for consistent reporting and missing data to be identified. The format was as follows: study characteristics, including number of patients, study design, patient characteristics and interventions received. Outcomes were grouped as tumour related outcomes and toxicity related outcomes.

## Results

### Quantity of the research

**Fig. 1:**
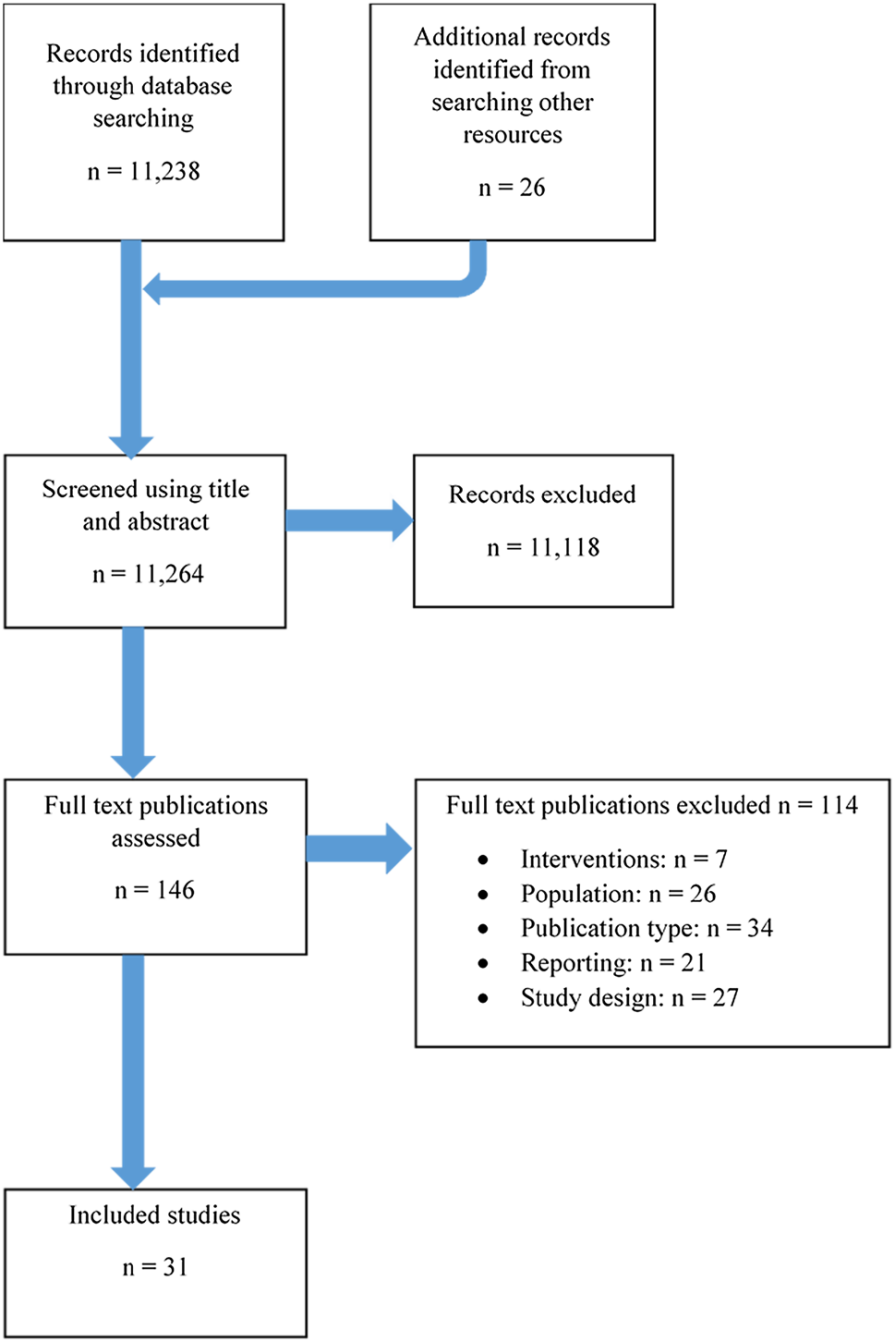
PRISMA diagram showing search process and number of included studies

Thirty-one full-text studies met the inclusion criteria, consisting of one phase II study, 24 retrospective and six prospective case studies. Twenty-three studies were single arm, the remaining were non-randomised comparisons of PBT with photon RT. There were no RCTs (Fig. 1).

Conducted in 10 institutions, 27 studies were based in the USA, one in France and two in Switzerland. One study was multinational with data from the USA and Canada [21]. In total, 1731 children participated in the studies, with 1465 children (85%) receiving PBT and 266 (15%) receiving photon RT. The studies were conducted between 1991 and 2018, with the majority of studies conducted between the years 2000 and 2015. The mean sample size was 51 and ranged from 10 to 179. Average follow-up ranged from 0.9 to 7.6 years (Table 1).

**Table 1:** Baseline characteristics of children and young adults with CNS tumours included in PBT studies

Study details Author, year [ref] Proton centre, years of study Country	Study design	N in study	Tumour type & location	Mean/median age in yrs (range) Male: n (%)	Disease stage & presence of metastasis	Extent of surgical resection %	Previous treatments	Mean follow-up (range)
Eaton (2016)[22] PBT: MGH 2000–2009 XRT: Emory 2000–2009 USA	Case series - retro comp	77 PBT: n = 40 [Shared PBT cohort with Yock(2016)[31]] XRT (3D-CRT /IMRT): n = 37	MB Standard risk: 100% Histology:- Classic: 82% Anaplastic or large cell variant: 7.7% Other: 10.3%	Median age at diag: PBT: 6.2 (3.3–21.9) XRT: 8.3 (3.4–19.5) Stat sign diff in ages P = 0.010 Male:- PBT: 21 (53%) XRT: 24 (65%)	ND: n = 77 (100%) Mets: 0% M0: 100%	Surgery: 100% Residual disease post- surgery: PBT:- None/GTR: n = 35 (88% < 1.5 cm^2^: n = 5 (12%) XRT:- None/GTR: n = 36 (97%) < 1.5 cm^2^: n = 1 (3%)	Surgery: 100% Adj Chemo: 100%	PBT: 5.8 yrs (3.4–9.9) XRT: 7.0 yrs (3.5–13.5)
Eaton (2016)[23] PBT: MGH 2000–2009: XRT: Emory 2000–2009 USA	Case series—retro comp	88 PBT: n = 45 [Shared PBT cohort With Yock (2016)[31]] XRT (3D-CRT /IMRT): n = 43	MB Standard risk: 100%	Median age at diag: PBT: 6.2 yrs ( 3.3 – 21.9) XRT: 8.2 yrs (3.4 – 19.5) Male: PBT: n = 2 5 (56%) XRT: n = 29 (67%)	Disease stage: NR Mets: 0%	Surgery: 100% Residual disease post-surgery: PBT: None/GTR: n = 40 (89%) < 1.5 cm^2^: n = 5 (11%) XRT: None/GTR: n = 42 (98%) < 1.5 cm^2^: n = 1 (2%)	Surgery: 100% Chemo: 100% (mainly adj)	Median (95% CI) for: PBT: 6.2 yrs (5.1 – 6.6) XRT: 7.0 yrs (5.8 – 8.9)
Grewal (2019)[24] Children’s Hospital 2010 – 2017 USA	Case series -retro non-comp	14	MB Age less than 60mths	Median age at diag: 29.1 mths (6 – 44mths) Median age at RT: 39.8 mths (10.9 – 62.9 mths) Male: n = 7pts (50%)	ND: n = 14 (100%) Mets: 0%	Surgery: 100% GTR: n = 10 STR: n = 4	Surgery: 100% Chemo: n = 13 (93%)	Median: 4.5yrs (1.5 – 7 yrs)
Jimenez (2013)[25] MGH 2002–2010 USA	Case series - retro non-comp	15	MB: n = 12 (80%) sPNET: n = 3 (20%)^a^	Mean age: 2.9 yrs (1.9–4.6) Male: n = 6 (40%)	ND: n = 15 (100%) Mets:- MB: n = 5 (33%) sPNET: n = 1 (7%)	Surgery: 100% MB (n = 12):- GTR: n = 10 (83%) STR: n = 2 (17%) sPNET (n = 3):- GTR: n = 1 (33.3%) STR: n = 1 (33.3%) Partial: n = 1 (33.3%) Second surgery: 7%	Pre-PBT: - Surgery: n = 15 (100%) - HDChemo. + ABMT (as per COG or Head Start protocol): n = 14 (93%)	3.25 yrs (0.25–8.5)
Kahalley (2019)[21] PBT: Texas Children’s 2007 – 2018 USA XRT: Hospital for Sick Children, 2007 – 2018 Canada	Case series -retro comp	79 PBT: n = 37; XRT: n = 42	MB PBT: Standard risk: n = 33 (78.6%) High risk: n = 9 (21.4%) RT: Standard risk: n = 24 (64.9%), High risk n = 13 (35.1%)	Mean age at diag: PBT: 8.4 yrs (3.6 – 15.5), XRT 8.9yrs (3.5 – 14.4) Male: PBT: 26(70%) XRT: 27(64)%	PBT: ND = 32 (86.5%), recurrent n = 5 (13.5%) XRT: ND = 36 (85.7%), recurrent n = 6 (14.3%)	Surgery: 100% Extent NR	Pre – PBT/XRT: according to protocol SJMB03 or SJMB12	Up to 10 yrs
Kamran (2018)[26] MGH 2002–2015 USA	Case series - prosp. non-comp	116	MB: n = 108 PNET: n = 8 Standard risk: n = 77 (66%) High risk: n = 39 (34%)	Median age: 7.6 yrs (2.1–18.1): ≤ 8: n = 60 (52%) > 8: n = 56 (48%) Male: n = 64 (55%)	NR	NR	NR	Median: 5 yrs (1–10.6) Mean: 5.03 yrs
Moeller (2011)[27] MDACC 2006–2009 USA	Case series - prosp. non-comp	23	MB Standard risk: n = 17 (74%) High-risk: n = 6 (26%)	Mean age: 6.0 yrs (3.0–16.0) Male: n = 14 (74%)	ND: n = 23 (100%) Mets: NR	Surgery: 100% (details of extent of resection NR)	Pre-PBT: Surgery: n = 23 (100%) Chemo: n = 6 (26%) Post-PBT: Chemo: n = 17 (74%)	0.9 yrs (0.7–1.3)
Paulino (2018)[28] PBT: MDACC 2007–2013 XRT: Texas Children’s 1997–2006 USA	Case series - retro comp	84 PBT: n = 38; XRT (3D-CRT + IMRT): n = 46	MB	Mean age (SD): PBT: 7.9 ± 3.4 Photon: 9.0 ± 4.0 Median (range): PBT: 7.6 (2.9–14.5) Photon: 9.0 (3.0–18.0) Male:- PBT: 28 (73.7%) Photon: 32 (69.6%)	NR	NR (all pts ‘underwent max safe resection’)	Pre-PBT/XRT: Surgery (maximally safe): n = 84 (100%) Post-PBT/XRT (4 wks): Chemo—Cisplatin: n = 84 (100%)	Median audiogram (end RT to last test) PBT: 4.7 yrs (1.1–8.4) XRT: 5.5 yrs (1.1–13.6)
Sethi (2014)[29] MGH 2002–2011 USA	Case series - retro non-comp	109	MB Standard risk: n = 74 (68%) High risk: n = 35 (32%) Histology:- Classic: 74% Anaplastic: 16% Desmoplastic: 9% Anaplastic & Desmoplastic: 1%	Mean age: 7.4 yrs (2.2–22.7) Male: n = 64 (59%)	ND: n = 109 (100%) Mets: n = 20 (18%)	Surgery: 100%:- GTR: n = 80 (73%) STR: n = 27 (25%) Biopsy: n = 2 (2%)	At diag (n = 109): Surgery: n = 109 (100%) Chemo: n = 109 (100%) At relapse (n = 16): Chemo: n = 12 (75%) Surgery: n = 4 (25%) Stereotactic radiosurgery n = 1 (7%) Focal fractionated RT: 12.5%	3.2 yrs (0.1–9.9)
Yock (2014)[30] PBT: MGH 2004–2009 XRT: Lucile Packard 2001–2002 USA	Case series - prosp. comp	48 PBT: n = 19 XRT (IMRT): n = 29	MB / sPNET^**b**^	Median age:- PBT: 10.0 yrs XRT: 10.0 yrs	NR	NR by tumour type	NR by tumour type	Median: PRT: 3.0 yrs XRT: 2.4 yrs
Yock (2016)[31] MGH 2003–2009 USA	Single arm phase II trial	59	MB Standard risk: n = 39 (66%) Intermediate risk: n = 6 (10%)^**c**^ High risk: n = 14 (24%) Histology:- Classic: n = 45 (76%) Desmoplastic or nodular variant: n = 6 (10%) Anaplastic or large cell variant: n = 8 (14%)	Median age: 6.6 yrs (5.1–9.9) Male: n = 33 (56%)	ND: n = 59 (100%) Mets: n = 14 (24%)	Surgery: 100%:- GTR / NTR: n = 55/58 (95%)	Pre-PBT: - Surgery: n = 58 (98%) - Standard-dose Chemo: n = 4 (7%) - HDChemo + ABMT: n = 2 (3%) Adj-Chemo: n = 52 (88%) Post-PBT:- Chemo: n = 57 (97%)	7 yrs (5.2–8.6)
Ares (2016)[32] CPTPSI 2004–2013 Switzerland	Case series -prosp. non-comp	50	Epend Intracranial: n = 50 (100%) Grade II: n = 4 (8%) Grade III: n = 46 (92%) Infratentorial: n = 36 (72%) Supratentorial: n = 14 (28%)	Median age at PBT: 2.6 yrs (1.1–15.2) 1–2 yrs: n = 13 (26%) 2–3 yrs: n = 18 (36%) 3–4 yrs: n = 10 (20%) 4–5 yrs: n = 3 (6%) > 5 yrs: n = 6 (12%) Male: n = 36 (72%)	NR	Surgery: n = 50 (100%):- GTR: n = 33 (66%) STR: ≤ 1.5 cubic centimetres (cc): n = 8 (16%) STR: > 1.5 cc: n = 9 (18%)	Pre-PBT: Surgery: n = 50 (100%) Second surgery: n = 11 (22%) Chemo: n = 43 (86%)	3.6 yrs (0.7–9.5)
Eaton (2015)[33] MGH 2004–2015 USA	Case series - retro non-comp **Pts undergoing re-irradiation**	20	Epend (intracranial; recurrent or mets) Infratentorial: n = 18 (90%) Supratentorial: n = 2 (10%) Histology:- Classic: n = 9 (45%) Anaplastic: n = 11 (55%)	Median age at re-irradiation: 5.3 yrs (3.3–23.8) Median age at diag: 2.1 yrs (0.9 – 19.5) Male: n = 8 (40%)	Recurrent/: n = 20 (100%)	Surgery:- At diag: n = 20 (100%):- GTR: n = 10 (50%) STR: n = 10 (50%) At progression: n = 20 (100%):- GTR: n = 8 (40%) STR: n = 7 (35%) None: n = 5 (25%)	Surgery: 100% At diag:- Chemo (pre-PBT): n = 8 (40%) Initial RT modality: PBTs: n = 17 (85%) Photons: n = 3 (15%) At relapse: Chemo (pre-PBT & post): n = 10 (50%), concurrent 2 (10%)	3.2 yrs (0.04–11.5)
Indelicato (2017)[34] UFCM 2007–2017 USA	Case series - prosp. non-comp	179	Epend (intracranial, non-mets) Tumour location:- Posterior fossa: n = 119 (66%) Supratentorial: n = 60 (34%) WHO Grade 2: n = 59 (33%) WHO Grade 3: n = 120 (67%)	Median age at first surgery: 3.0 years (0.4- 20.6); Median age at PBT: 3.5 years (0.7- 21.3); Pts aged ≤ 3 years: n = 98 (55%) Male: n = 103 (58%)	Recurrent: n = 18 (10%) Mets: 0%	Surgery: 100%:- GTR/NTR: n = 152 (85%)	Pre-PBT: Surgery: 100% [Number of operations:- 1: n = 131 (73%) 2: n = 40 (22%) 3 + : n = 8 (5%)] Chemo(pre-PBT): n = 59 (33%), 36 (20%) of which received high-dose methotrexate No prior radiation	Median: 3.2 yrs (0.1 to 9.6)
MacDonald (2013)[35] MGH 2000–2011 USA	Case series - retro non-comp (consecutive recruitment)	70	Epend Supratentorial: n = 19 (27%) Infratentorial: n = 51 (73%) Histology:- Differentiated classic: n = 37 (53%) Anaplastic: 33 (47%)	Mean age: 3.2 yrs (0.25 -20) Male: n = 33 (47%)	ND: n = 65 (93%) Recurrent: n = 5 (7%) Mets: 0%	Surgery:-100%: GTR: n = 46 (66%) NTR: n = 1 (1%) STR: n = 23 (33%) Number of surgeries:- 1: n = 54 (77%); 2: n = 14 (20%); 3: n = 2 (3%) Shunt: n = 29 (76%) pts with hydrocephalus	Pre-PBT: Surgery: n = 70 (100%) Chemo: n = 21 (30%)	3.8 yrs (1.0–11.7)
Sato (2017)[36] PBT: MDACC 2006–2013 XRT: Texas Children’s 2000–2009 USA	Case series - retro comp	79 PBT: n = 41 XRT (IMRT): n = 38	Epend (localised) WHO grade II (differentiated):- - PBT: n = 8 (20%) - XRT: n = 7 (18%) WHO grade III (anaplastic): - PBT: n = 33 (80%) - XRT: n = 31 (82%) Infrantentorial:- - PBT: n = 31 (76%) - XRT: n = 23 (61%)	Median age at diag: PBT: 2.5 (0.5–18.7); < 3 yrs: n = 7 (66%) XRT: 5.7 yrs (0.4–16.5); < 3 yrs: n = 11 (29%) Male: PBT: n = 25 (61%) Photon: n = 21 (55%)	ND: n = 79 (100%) Mets: 0%	PBT:- Surgery: n = 41 (100%); GTR: n = 38 (93%) XRT:- Surgery: n = 38 (100%) GTR: n = 29 (76%)	Pre-RT:- Surgery: n = 79 (100%) Chemo(for pts achieving STR/young age [< 1 year] at diag): All pts: n = 15 (19%):- PBT: n = 6 (15%) XRT: n = 9 (24%)	Median:- PRT: 2.6 yrs (0.6–7.2) XRT: 4.9 yrs (1.1–11.7)
De Amorim Bernstein (2013)[37] MGH 2004–2011 USA	Case series - retro non-comp	10	AT/RT Tumour location:- Supratentorial: n = 6 (60%) Infratentorial: n = 3 (30%) Brachial plexus: n = 1 (10%)	Mean age: 1.8 yrs (15 days-19.3) Male: n = 2 (20%)	ND: n = 10 (100%) Mets: 0%	Surgery: n = 10 (100%):- GTR: n = 4 (40%) NTR: n = 4 (40%) STR: n = 2 (20%)	Surgery: n = 10 (100%) Induction SDChemo: n = 10 (100%) Post-PBT:- Chemo ± HDChemo + ABMT or intrathecal Chemo: n = 10 (100%)	2.3 yrs (0.9–8.3)
Haskins (2015)[38] IUSM 2007–2013 USA	Case series - retro non-comp	16	AT/RT	Mean age: 1.5 yrs (0.4–39) Male: n = 12 (75%)	ND: n = 16 (100%) Mets: n = 5 (31%)	Surgery: n = 14 (88%):- GTR: n = 8 (50%) STR: n = 6 (38%) None: n = 2 (12%)	Pre-PBT:- Surgery: n = 14 (88%) Chemo or HDChemo: n = 15 (94%) Post-PBT:- Consolidation HDChemo + ABMT: n = 15 (94%)	3.18 yrs
McGovern (2014)[50] MDACC 2008–2013 USA	Case series - retro non-comp	31	AT/RT Tumour location:- Primary site in the brain: n = 16 (52%) Stage:- M1: n = 3 (10%) M2: n = 5 (16%) M3: n = 6 (19%) Synchronous kidney disease: n = 1 (3%)	Mean age: 2.0 yrs (0.5–5.2) Male: n = 13 (42%)	ND: n = 31 (100%) Mets: n = 15 (48%)	Surgery: n = 31 (100%):- GTR: n = 15 (48%) STR: n = 13 (42%) Biopsy: n = 3 (10%) Second-look surgery (pre-RT): n = 2 (6%)	Surgery/biopsy: n = 31 (100%) Chemo:- Pre-PBT: n = 26 (84%) Concurrent with PBT: n = 11 (35%) Post PBT: n = 17 (55%)	2.0 yrs (0.25–4.4)
Weber (2015)[40] CPTPSI 2008–2013 Switzerland	Case series - retro non-comp	15	AT/RT (non-mets) INI-1 loss: n = 15 (100%) Tumour location:- Supratentorial: 40% Posterior fossa: 60%	Mean age: 1.5 yrs (0.9 – 2.1) Male: n = 8 (53%)	ND: n = 15 (100%) Mets: 0%	Surgery: n = 15 (100%):- GTR: n = 7 (47%) STR: n = 7 (47%) Biopsy: n = 1 (6%) Second surgery (post-chemo): n = 3 (20%)	Surgery: n = 15 (100%) Chemo:- Pre-PBT: n = 15 (100%) Concurrent with PBT: n = 7 (47%) Salvage therapy: n = 6 (40%)	2.8 yrs (0.8 – 5.8)
Bass (2018)[41] SJCRH 2011–2016 USA	Case series -prosp. non-comp	74	Cranio	Median age start of PBT: 10 yrs (4.0—19.3) Male: n = 35 (48%)	NR	Surgery: 74 (100%): GTR: n = 0 STR: n = 74 (100%)	Surgery: n = 74% (100%)	2.0 yrs (1.0 – 5.0)
Bishop (2014)[42] PBT: MDACC 2007–2012 XRT: Methodist Hospital 1996–2007 USA	Case series - retro comp	52 PBT: n = 21 (40%) XRT (IMRT): n = 31 (60%)	Cranio	Median age: 8.9 yrs (range: NR) Male:- PBT: n = 9 (43%) Photon: n = 14 (45%)	ND: n = 30 (58%) Recurrent: n = 22 (42%) ND:- PBT: n = 12 (57%) XRT: n = 18 (58%) Recurrent: PBT: n = 9 (43%) XRT: n = 13 (42%)	PBT:- Surgery: n = 21 (100%):- GTR: n = 5 (24%) STR: n = 9 (43%) Other (cyst drainage, fenestration, shunting): n = 7 (33%) XRT:- Surgery: n = 31 (100%) GTR: n = 1 (3%) STR: n = 11 (36%) Other: n = 19 (61%) (P = 0.032)	Surgery:- Number of surgeries: One:- PBT: n = 15 (71%) XRT: n = 17 (55%) Two:- PBT: n = 4 (19%) XRT: n = 9 (29%) Three:- PBT: n = 2 (10%) XRT: n = 4 (13%) Four:- PBT: 0% XRT: n = 1 (3%)	PBT: 2.8 yrs (0.9–5.5) XRT: 8.8 yrs (0.7–15.4)
Jimenez (2021)[43] MGH 2002–2018 USA	Case series – retro non-comp	77	Cranio	Mean age diag: 8.6 yrs (1.3 – 20yrs) Mean age RT: 9.6yrs ( 2.3—20.5yrs) Male n = 41pts (53%)	ND n = 30 (39%) Recurrent: n = 47(61%)	Surgery: n = 70 (91%) GTR: 14pts (18%) STR: 46 pts (60%)	STR: n = 46 (60%) Biopsy/cyst fenestration n = 17 (22%) GTR n = 14 (18%)	4.8 yrs (0.8 – 15.6)
Laffond (2012)[44] ICPO 1995–2007 France	Case series - retro non-comp	29	Cranio	Mean age at diag: 7.8 yrs (SD 4.1); Median: 7 yrs (range 1.8 to 15.8) Mean age during study: 14 yrs (SD 4.1); Median: 13.8 yrs (7.1—24) Male: n = 15 (52%)	ND: n = 18 (62%) Recurrent: n = 11 (38%) Mets: 0%	Surgery: n = 28 (97%):- GTR: n = 6 (21%) STR or partial resection: n = 21 (76%) Number of resections:- 1: n = 18 (64%); 2: n = 5 (18%); 3: n = 3 (11%); 4: n = 2 (7%) Ommaya reservoir: n = 1 (3%)	Surgery: n = 97%	6.2 yrs (SD: 4.5) HRQoL: 4.1 yrs (1.7–14.0)
Luu (2006)[45] LLUMC 1991–2000 USA	Case series—retro non-comp	16	Cranio	Average age: NR Age range at diag: 3.0 -17.0 yrs Male: n = 10 (63%)	ND: n = 4 (25%) Recurrent: n = 12 (75%)	Surgery: n = 16 (100%):- Extent of resection: NR Number of resections:- 1 resection: n = 9 (56%) > 1 resection: n = 7 (44%)	Surgery: N = 16 (100%):- 1 resection: + PBT (initial Adj treatment): n = 4 (25%) 1 resection + salvage PBT: n = 5 (31%) > 1 resection, repeat resection + Adj PBT: n = 7 (43%)	5.0 yrs (1–10)
Winkfield (2009)[46] MGH 2001–2007 USA	Case series - retro non-comp	24	Cranio No cystic component: n = 5 (21%) Cystic component: n = 19 (79%)	Mean age: 8.4 yrs (3.0 -14.0) Male: n = 14 (58%)	ND: n = 18 (75%) Recurrent: n = 6 (25%)	Surgery: n = 24 (100%):- GTR: n = 4 (17%) STR: n = 16 (66%) Biopsy with cyst drainage: n = 4 (17%) Second surgery: n = 7 (28%) after relapse Third surgery after relapse: n = 2 (8%)	Surgery No further additional treatments reported	3.4 yrs (0.5–6.5)
Greenberger (2014)[47] MGH 1995–2007 USA	Case series - retro non-comp	32 NB: Pts required to have at least 3-years follow-up to be eligible for the study	LGG WHO grade I (JPA): n = 19 (59%) WHO grade II: n = 6 (19%) Low grade (not specified): n = 2 (6%) No pathology: n = 5 (16%) Supratentorial: n = 18 (56%) Infratentorial: n = 11 (34%) Spinal: n = 3 (9%)	Median age: at diag: 7.6 yrs (0.8–20.4) at treatment: 11.0 yrs (2.7–21.5) Male: n = 17 (53%)	ND: n = 32 (100%) Mets: NR	Surgery: n = 21 (66%):- Biopsy: n = 6 (18%) Resection: n = 21 (66%):- number of resections: n = 1: n = 17 (53%) n = ≥ 2: n = 4 (13%) Shunt(s): n = 6 (19%)	Pre-PBT:- Surgery: n = 21 (66%) Chemo: n = 16 (50%):- No. of chemo regimens: 1: n = 6 (19%); 2: n = 7 (22%); 3: n = 3 (9%)	Survival outcomes: 7.6 yrs) (3.2–18.2) Neuro-cog: 4.8 yrs (1.2–8.1) Endocrine 20yrs
Hug (2002)[48] LLUMC 1991–1997 USA	Case series - retro non-comp	27	LGG Diffuse low-grade astrocytoma: n = 9 (33%) JPA: 52% No tissue diag: n = 5 (19%) (Optic pathway glioma)	Mean age at time of PBT: 8.7 yrs (2–18) Male: n = 14 (52%)	ND: n = 12 (44%) Recurrent: n = 15 (56%) Mets: NR	Complete radiographic resection: n = 1 (4%) Complete resection but residual enhancement: n = 1 (4%) STR: n = 21 (77%) No resection (biopsy only or radiographic diag): n = 4 (15%)	Surgery No further additional treatments reported	3.3 yrs (0.6–6.8)
Indelicato (2019)[49] UFCM 2007–2017 USA	Case series—prosp. non-comp	174	LGG: - Pilocytic astrocytoma: n = 81 (47%) - Ganglioglioma: n = 15 (9%) - Grade 1, other: n = 5 (3%) - Diffuse astrocytoma: n = 19 (11%) - Pilomyxoid astrocytoma: n = 16 (9%) - Oligodendroglioma: n = 8 (5%) - Pleomorphic xanthroastrocytoma: n = 2 (1%) - Grade 2, other: n = 6 (3%) - No biopsy: n = 22 (12%) Tumour location:- - Diencephalic/optic pathway: n = 90 (52%) - Caudal brainstem: n = 28 (16%) - Cerebellum: n = 22 (13%) - Cerebral hemisphere: n = 16 (9%) - Tectum: n = 10 (6%) - Spinal cord: n = 8 (4%)	Median age during PBT:- Overall: 9 yrs (2–21); 26 pts (15%) < 6 yrs old Children receiving initial chemo: 7 yrs (2–19 yrs) Children not receiving initial chemo: 12 yrs (3 -21) Male: n = 88 (51%)	WHO grade I: 122 (70%) WHO grade 2: 52 (30%) Mets: 0%	Surgery: n = 152 (87%):- Gross total resection (GTR): n = 5 (3%) Sub-total resection (STR)/biopsy: n = 147 (84%) No surgery: n = 22 (13%)	Median interval between diag &start of radiation: 2.7 years (range, 0.1–18.4) • Surgery: n = 152 (87%) • Chemo therapy: n = 74 (43%) - mostly carboplatin/vincristine: n = 51 (29%); - forty-five pts (26%) received > 1 chemo regimens (maximum = 9 different chemo regimens before RT): - 0 regimens: n = 100 (57%) - 1 regimen: n = 29 (17%) - 2 regimens: n = 23 (13%) - 3 + regimens: n = 22 (13%)	Median: 4.4 yrs
MacDonald (2011)[50] MGH 1998–2007 USA	Case series - retro non-comp	22	Germ cell tumours Germinoma: n = 13 (59%) NGGCT: n = 9 (41%) Tumour location:- Pineal gland: n = 4 (18%) SUP: n = 10 (46%) Multiple midline lesions: n = 6 (27%) Multiple sites of brain: n = 2 (9%)	Mean age: 11.0 yrs (6.0- 20.0) Male: n = 11 (50%)	ND: n = 22 (100%) Mets: 0%	NR	Surgery: post-chemo/pre-PBT: 11% Germinoma:- Chemo: n = 11 (85%) NGGCT:- Chemo: n = 9 (100%)	2.3 yrs (1.1- 8.1)
Farnia (2014)[51] MDACC PBT 2007–2012 XRT 1982 – 2005 USA	Case series - retro comp. [22]	11 PBT 10 XRT	Pineo Tumour location:- Pineal gland	Mean age:- PBT: 7.7 yrs (0.3–20.0) XRT:14.5 yrs (3.8–21.9) Male: PBT 3/11 (27%) XRT 4/10 (40%)	NR Mets: 0%	PBT: Surg: n = 10/11 (91%):- GTR: n = 6/10 (60%) STR: n = 4/10 (40%) XRT: Surg: n = 7/10 (70%):- GTR: n = 2/7 (29%) STR: n = 5/7 (71%)	Surgery: 81% Chemo: 100% (either before, concurrent or post-PBT)	3.2 yrs (0.2–27.7)

**Key: a:** 2016 WHO Classification of Tumours of the Central Nervous System sPNETs are now viewed as a mix of tumours of other lineages, such as AT/RT, astrocytic tumours & a few newly defined entities; **b:** This study included pts with other CNS tumour types, only MB/PNET had sufficient patient numbers [(PBT: n = 19 (33%; Photon RT: n = 29 (46%)] to meet the inclusion criteria (see p.4); **c:** Post-2008:- Intermediate risk group defined as M0 disease with minimal or no residual disease with anaplastic or large cell variant histology; in Intermediate risk pts analysed for survival endpoints with high-risk pts as well as separately.

*3D-CRT* Three-dimensional conformal radiation therapy, *Adj* adjuvant, *AT/RT* Atypical Teratoid/Rhabdoid Tumour, *Chemo* chemotherapy (standard dose), *comp* comparative, *Cranio* craniopharyngioma, *Diag* diagnosis, *Epend* ependymoma, *GCT* germ cell tumour, *GTR* gross total resection, *HDChemo + ABMT* high dose chemo plus autologous bone marrow transplant, *HRQoL* health-related quality of life, *IMRT* Intensity Modulated Radiation Therapy, *JPA* Juvenile Pilocytic Astrocytoma (WHO grade I), *MB* medulloblastoma, *ND* newly diagnosed, *NGGCT* Non-Germinomatous Germ Cell Tumour, *NR* not reported, *NTR* near total resection, *Pineo* pineoblastoma, *PBT* proton beam therapy, *PF* posterior fossa, *prosp* prospective, *pts* patients, *STR* sub-total resection, *(s)PNET* (supratentorial) primitive neuroectodermal tumour, *retro* retrospective, *Un* unclassified, *WHO* World Health Organisation, *XRT* photon radiotherapy

*Proton centres* CPTPSI Center for Proton Therapy, Paul Scherrer Institute, Switzerland, *Children’s Hospital* Children’s Hospital Pennsylvania USA, *ICPO* Institut Curie Proton Beam Therapy Centre, Orsay, France, *IUHPTC* Indiana University Health Proton Therapy Center, Indiana, USA, *IUSM* Indiana University School of Medicine, Indianapolis, USA, *LLUMC* Loma Linda University Medical Centre, California, USA, *MDACC* University of Texas MD Anderson Cancer Centre, Houston,TX, USA, *MGH* Massachusetts General Hospital, Boston, Mass, USA, *SJCRH* St. Jude Children’s Research Hospital, Memphis, TN, USA, *UFCM* University of Florida College of Medicine, Gainesville, FL, USA

Eleven studies included children with medulloblastoma/primitive neuroectodermal tumours (PNET) (n = 712) [21–31], five ependymoma (n = 398) [32–36], four atypical teratoid/rhabdoid tumour (AT/RT) (n = 72) [37–40], six craniopharyngioma (n = 272) [41–46], three low-grade glioma (LGG) (n = 233) [47–49], one germ cell tumours (GCT) (n = 22)[50], and one pineoblastoma (n = 22) [51]. Ninety percent of patients were receiving first-line therapy and 57% were male (Table 1).

### Quality of the research

Selection bias and reporting bias were the major methodological limitations, due to studies involving opportunity/convenience samples and the retrospective nature of the data collection. Poor reporting compounded selection bias with few studies reporting eligibility criteria making it difficult to assess representativeness and generalisability. Where studies included patients at different stages in disease progression, most did not report results separately by disease status. Poor reporting also hampered assessments of outcomes, for example, timing of outcome assessments was generally not reported and long-term adverse events were frequently reported in a seemingly arbitrary sub-group of patients. Length of follow-up was long enough for some outcomes to occur (e.g. PFS in AT/RT), but not others (e.g. long-term adverse events, particularly neuro-cognitive outcomes) (SI Fig. 1).

### Medulloblastoma

Eleven studies assessed the effects of PBT, reporting data on 712 patients with medulloblastoma/PNET, with 515 receiving PBT and 197 receiving photon RT. In seven studies children were treated with PBT at the Massachusetts General Hospital (MGH). All MGH studies have slightly different study designs and focus, but it should be noted that double counting for common outcomes may have occurred as there is substantial overlap in study dates/periods suggesting a shared cohort of patients particularly between 2002 and 2009 and for OS outcomes.

The 11 studies comprised of one single-arm phase II trial [31] and 10 case series studies (three prospective [26, 27, 30] and seven retrospective [21–25, 28, 29]. Five studies compared PBT (n = 179) with photon RT (n = 197) [21–23, 28, 30]. The mean sample size was 65. Median follow-up ranged from 0.9 to 7 years. One study had 11 (14%) recurrent patients [21].

Eight studies defined patients according to risk, with 78% (429/551) defined as standard-risk and 21% (115/551) defined as high-risk. One study defined six patients as intermediate-risk—see paper for definitions—accounting for 1% of the total, however, these patients outcomes are reported as if they were high-risk [31]. Across the studies the youngest patient was 1.9 years [25], the oldest 21.9 years [22] but the median age within the studies ranged from 2.9 to 10 years. Two studies focused solely on very young children [24, 25] (Table 1).

PBT was given as part of a multimodal treatment regimen consisting of surgical resection prior to radiotherapy and chemotherapy (various protocols). Gross total resection (GTR) was achieved in 86% of PBT patients. The median craniospinal irradiation (CSI) dose for standard-risk patients was 23.4 Gy_RBE_ (36.0 Gy_RBE_ for high-risk patients) with a median boost dose to the tumour bed of 54 Gy_RBE_ both delivered in fractions of 1.8 Gy_RBE_. (Table 1 and SI Table 1).

#### Tumour related outcomes

Survival was reported in five studies (n = 285) [23–25, 29, 31]. OS for all PBT patients ranged from 68 to 89% in newly diagnosed patients, depending on patient and tumour characteristics and follow-up. For example, Yock (2016) reported 7-year OS rates of 81% for 39 standard-risk PBT patients compared with 68% for 20 high-risk PBT patients [31]. Eaton (2016) reported a 6-year OS of 82% for 45 PBT patients compared with 88% for 43 photon RT patients but the comparison was non-significant [23]. In very young children, Grewal reported an OS of 84% at 5 years in 14 PBT patients [24] (Table 2).

**Table 2:** Summary of results of tumour related outcomes studies on PBT in children and young adults with CNS tumours

Study details Author, year [ref]	Tumour type	N	Overall Survival	Progression-Free Survival	Time to Progression	Response Rates	Local Failure Rate	Distant Failure Rate
Eaton (2016)[23]^a^ Shared PBT cohort with Yock 2016[31]	MB	88 PBT: 45 XRT: 43	6 yrs:- PBT: 82.0% (95% CI: 65.4–91.1) XRT: 87.6% (95% CI: 72.7–94.7) (p = 0.285)	6 yr RFS:- PBT: 78.8% (95% CI: 63–89) XRT: 76.5% (95% CI: 60.6–86.6) (p = 0.948) 10 pts in each cohort experienced a recurrence (p = 0.908)	NR	NR	NR	NR
Grewal (2019)[24]	MB	14	OS: 1 yr 93% (KM) OS: 5 yr 84% (95% CI 48 0 96%)	RFS: 1 yr 86% RFS: 5 yrs 70% (95% CI 48–96%)	Median time to relapse post RT: 0.9 yr	NR	NR	Progression outside posterior fossa = 3pts (42%)
Jimenez (2013)[43]^b^	MB /sPNET	15	3.25 yrs: 85.6% (95% CI: 68.6–100)	NR	NR	NR	Local failure rate = 1 pts at 12 months	No distant failure reported
Sethi (2014)[29]^d^	MB	109	3.2 yrs: 89%	NR	Median: 1.55 yrs (0.23–3.24)	NR	3.2 yrs: 5%	3.2 yrs: 10%
Yock (2016)[31]^b^	MB	59	All pts:- 5.0 yrs: 83% (95% CI: 70–90) 7.0 yrs: 81% (95% CI: 67–89); Standard-risk pts (n = 39; 66%):- 5.0 yrs: 86% (95% CI: 70–94) 7.0 yrs: 86% (95% CI: 70–94); Intermediate-high risk pts (n = 20; 34%):- 5.0 yrs: 75% (95% CI: 50–89) 7.0 yrs: 68% (95% CI: 42–84)	All pts: 5.0 yrs: 80% (95% CI: 67–88) 7.0yrs: 75% (95% CI: 61–84) Standard-risk pts (n = 39; 66%):- 5.0 yrs: 85% (95% CI: 69–93) 7.0 yrs: 81% (95% CI: 64–91) Intermediate-high risk pts (n = 20; 34%):- 5.0 yrs: 70% (95% CI: 45–85) 7.0 yrs: 63% (95% CI: 37–81)	Standard-risk pts (n = 39; 66%):- median 2.5 yrs (1.3–4.4) Intermediate-high risk pts (n = 24; 34%):- median 1.3 yrs (0.8–2.3)	NR	NR	NR
Ares (2016)[32]^b^	Epend	50	5.0 yrs: 84% (SD ± 6.8%); Five pts (10%) died of progressive disease due to local or distant failure at a median time of 2.75 yrs (range 2–4)	NR	NR	Of 17 pts who achieved an STR: CR: n = 13 (76%) PR/SD: n = 3 (18%) PD: n = 1 (6%) All 17 pts progressed at a mean interval of 19 mths (range 9–16 mths)	5.0 yrs: 22%	2/ 50 4% (no time given)
Eaton (2015)[33]^c^	Epend	20	3.0 yrs: 76% (95% CI: 67.6–89.6)	Median: 1.6 yrs (95% CI: 1.0–2.2) 1.0 yrs: 66.5% (95% CI: 55.2–77.8) 3.0 yrs: 28.1% (95% CI: 15.6–40.6)	NR	NR	3.0 yrs**:** 5/11 (45%) pts with a first local failure Failure rates directly related to pattern of first failure	3.0 yrs: 6/9 (67%) pts first distant failure Failure rates directly related to pattern of first failure
Indelicato (2017)[34]^d^	Epend	179	3.0 yrs: 90.4%	3.0 yrs 75.9%	NR	NR	3 yrs: 14.6% (95% CI: 9.6–21.7) Median time to local failure was 1.4 yrs (range, 0.2–2.5)	3 years: 15.4% (95% CI: 10.4–22.2) Median time to distant failure was 1.0 yrs (range, 0.2–6.1)
MacDonald (2013)[35]^b^	Epend	70	3.0 yrs: 95% (95% CI: NR)	3.0 yrs: 76% (95% CI: NR)	NR	NR	3.0 yrs: 17% 5.0 yrs: 23%	3.0 yrs: 14% 5.0 yrs: 17%
Sato (2017)[36]^a^	Epend	79 PBT: 41 XRT: 38	3.0 yrs: PBT: 97% (95% CI: 83–99) XRT: 81% (95% CI: 63–90) (P = 0.08)	3.0 yrs PFS^e^: PBT: 82% (95% CI: 64–92) XRT: 60% (95% CI: 42%-74%) (P = 0.031) 3.0 yrs local RFS^e^:- PBT: 88% XRT: 65%	NR	NR	PBT: n = 6 (15%) at 2.6 yrs median follow-up XRT: n = 18 (47%) at 4.9 yrs median follow-up	PBT: n = 1 (2%) at 2.6 yrs median follow-up XRT: n = 3 (8%) at 4.9 yrs median follow-up
De Amorim Bernstein (2013)[37]^b^	AT/RT	10	2.3 yrs – last fu: 90%	NR	NR	CCR: n = 6 (70%) CR: n = 2 (20%) SD: n = 1 (10%)	2.3 yrs: 0%	2.3 yrs: n = 2 (20%)
Haskins (2015)[38]^c^	AT/RT	16	Mean: 5.6 years (95% CI: 4.4–6.9) 3.2 yrs: 81% (95% CI: NR)	1.4 yrs: 75% (95% CI: NR)	NR	1.4 yrs:- NED: n = 6 (38%) SD: n = 7 (44%) DOD: n = 3 (18%) PD: 6% (Secondary malignancy)	3.2 yrs: n = 2 (13%)	2.3 yrs: n = 4 (26%)
McGovern (2014)[39]^a,c^	AT/RT	31	Median OS from diag: 2.9 yrs; 2.0 yrs from diag: 68.3% (95% CI: 53.9–8.1); 2.0 yrs from end of PBT: 52.9% (95% CI: 36.0–77.8)	Median PFS from diag: 1.7 yrs; 2.0 yrs from diag: 47.6% (95% CI: 32.2–70.5); 2.0 yrs from end of PBT: 45.9% (95% CI: 29.4–71.4)	NR	NR	NR	NR
Weber (2015)[40]^a^	AT/RT	15	2.0 yrs: 64.6% (95% CI: 39–90)	2.0 yrs: 66% (95% CI: 42–90)	NR	N = 7 (residual disease prior to PBT): CR: n = 2 (29%) SD: n = 3 (42%) PD: n = 2 (29%)	2.8 yrs: n = 3 (20%)	2.8 yrs: n = 4** (**27%)
Bishop (2014)[42]^c^	Cranio	52	3.0 yrs (whole cohort): 96% 3.0 yrs (PBT vs XRT): 94.1% vs 96.8% (p = 0.74)	NR	NR	NR	NR	NR
Luu (2006)[45]^d^	Cranio	16	5.0 yrs (1 resection): 100%; 5.0 yrs (> 1 resection): 60%	NR	NR	NR	5.0 yrs: n = 1 (6%)	NR
Jimenez (2021)[43]^b^	Cranio	77	5.0 yrs: 97.7% (95%CI 84.6 – 99.7%)	NR	NR	NR	5.0 yrs: 9.9% (95% CI 3.5 – 20.2%) Median fu: 4.8yrs (range 0.8 – 15.6) 6 pts had LF Median time to failure 3.6yrs (range 1.8 – 8.4) from RT end	NR
Winkfield (2009)[46]	Cranio	24	NR	NR	NR	NR	3.4 yrs: 0%	NR
Greenberger (2014)[47]^b^	LGG	32	8 yrs: 100% (95% CI: NR)^f^	6.0 yrs: 90% (95% CI:NR) 8.0 yrs: 83% (95% CI:NR)	NR	NR	NR	NR
Hug (2002)[48]^c^	LGG	27	3.3 yrs: 85% (95% CI: NR)	NR	NR	NR	3.3 yrs: 22%	3.3 yrs: 0%
Indelicato (2019)[49]^d^	LGG	174	5.0 yrs: 92% (95% CI, 85%-95%)	5.0 yrs: 84% (95% CI, 77%-89%)	Median time to LF: 1.0 yr (0.3–4.4)	NR	5.0 yrs: 15%	NR
MacDonald (2011)[50]^d^	GCT	22	2.3 yrs: 100%	2.3 yrs: 95% (95% CI: NR)	NR	NR	2.3 yrs: Germinoma: 0% NGGCT: 0%	2.3 yrs: Germinoma: 0% NGGCT: n = 1 (11%)
Farnia (2014)[51]^a^	Pineo	22	3.2 yrs^g^: 90%^h^ PBT: 88% (2 deaths) PHOT: 45% (6 deaths)	NR	NR	NR	1.7 yrs: 10%	1.4 yrs: 10%

**Key**:- a: survival outcomes measured from the time of diag; b: survival outcomes measured from the start of PBT; c: survival outcomes measured from completion of PBT; d: time survival outcomes measured from not reported; e: PFS and RFS values approximate as not reported directly in study but extrapolated from Kaplan Meier curves by reviewer; f: patients required to have at least 3-year follow-up to be eligible for study; g: study also included 21 adult patients but survival outcomes and adverse event data not reported separately for children; h: not calculated according to Kaplan–Meier method but rather 9/10 patients alive at time of analysis.

*CCR* continuing complete response, *CR* complete response, *Cranio* Craniopharyngioma, *DOD* died of disease, *Epend* Ependymoma, *GCT* Germ Cell Tumour, *LGG* Low Grade Gliomas, *MB* Medulloblastoma, *NED* no evidence of disease, *NGGCT* Non-Germinomatous Germ Cell Tumour, *NR* not reported, *PBT* proton beam therapy, *PD* progressive disease, *PIN* Pineoblastoma, *Photon RT* photon radiotherapy, *PR* partial response, *Pineo* pineoblastoma, *pts* patients, *RFS* recurrence-free survival, *RR* response rate, *SD* Stable disease, *(s)PNET* (supratentorial) Primitive Neuroectodermal Tumour

Failure rates were given in three studies for PBT patients [24, 25, 29]. At 3.2 years, LFR was 5% and DFR 10% (n = 109), with the spine the most common site for isolated local failure (Table 2).

#### Toxicity related outcomes

Early to medium term toxicities were reported in two studies [24, 31]. Serious adverse events experienced 90-days post PBT included stroke (grade IV) in one patient and brainstem injury consistent with necrosis (grade III) in another, with no toxicity-related deaths reported [24, 31]. One patient died from viable tumour and necrosis in the brainstem, but it was unclear if the necrosis was related to PBT [24] (Table 3).

**Table 3:** Adverse events other than endocrinopathies, ototoxicities or neuro-cognitive outcomes

Author, year [ref]	Tumour type	Adverse events	Follow-up / Reported at
Grewal (2019)[24]	MB	Mixed viable tumour & necrosis within brainstem n = 1 (this pt was one of the children who died)	0.8 yrs
Yock (2016)[31]	MB	**Acute toxic effects**^**a**^**, based on 59 pts:** Alopecia grade II: n = 59 (100%); fatigue grade I: n = 22 (37%), grade II: n = 18 (13%), grade II: n = 5 (8%); anorexia grade I: n = 14 (24%), grade II: n = 14 (24%), grade III: n = 7 (12%); nausea grade I: n = 25 (42%), grade 2: n = 7 (12%), grade III: n = 2 (3%); radiation dermatitis grade I: n = 44 (75%), grade II: n = 12 (20%), grade III: n = 2 (3%); oesophagitis, pharyngitis, or dysphagia grade I: n = 9 (15%), grade II: n = 9 (15%), grade III: n = 3 (5%); headache grade I: n = 13 (22%), grade II: n = 4 (7%); weight loss grade I: n = 6 (10%), grade: n = 4 (7%); Neutropenia grade I: n = 1 (2%), grade II: n = 22 (37%), grade III: n = 19 (32%), grade IV: n = 5 (8%); anaemia (haemoglobin) grade I: n = 10 (17%), grade II: n = 28 (47%), grade III: n = 3 (5%); lymphopenia grade II: n = 6 (10%), grade III: n = 10 (17%), grade IV: n = 7 (12%); thrombocytopenia grade I: n = 10 (17%), grade II: n = 1 (2%), grade III: n = 2 (3%) **Late toxic effects**^**a**^**, based on 58 pts:** Stroke grade IV – survived: n = 1 (2%); cataracts grade I: n = 11 (19%), grade II: n = 1 (2%), grade III: n = 4 (8%); obesity grade II: n = 5 (10%); grade III: n = 2 (4%); alopecia grade I: n = 16 (27%), grade II: n = 4 (7%); CNS brainstem injury grade III consistent with necrosis—survived: n = 1 (2%); ataxia grade I: n = 24 (41%), grade II: n = 4 (8%); headaches grade I: n = 7 (12%), grade II: n = 4 (7%); dysphasia grade I: n = 3 (5%), grade II: n = 2 (4%); chronic fatigue grade I: n = 5 (9%), grade II: n = 2 (4%); depression grade I: n = 2 (3%), grade II: n = 2 (4%); scoliosis (present at radiotherapy) grade I: n = 4 (7%), grade II: n = 1 (2%); truncal muscle weakness grade II: n = 1 (2%); & nystagmus grade I: n = 10 (17%) No treatment related deaths	Acute effects: ‘occurred up to 90-days post-completion of PBT’ Late effects: ‘occurred after 90-days post-completion of PBT’ CNS brainstem injury occurred in a 14 year old boy 6.9 yrs post irradiation
Ares (2016)[32]	Epend	Based on 50 (100%) pts: grade 1 patchy alopecia or hair thinning: n = 7 (14%); Grade 1 concentration problems: n = 1 (2%); grade 1 asymptomatic transient MRI changes of leukoencephalopathy: n = 9 (18%); fatal brainstem – possibly due to second surgery where there was a brainstem infarct & meningitis: n = 1 (2%)	3.6 yrs
Eaton (2015)[33]	Epend	3/14 (21%) Grade II radiation-associated toxicity experienced in local re-treated pts: n = 3/14 (21%); headache (n = 1; 7%); cranial nerve VI palsy (n = 1; 7%); neck pain (n = 1; 7%)	0.2—2.7 yrs post-re-irradiation
Indelicato (2017)[34]	Epend	Based on 179 (100%) pts:—short term toxicity = 18 (10%) had nausea/vomiting; 1 (0.6%) headache requiring opioid analgesia; longer term toxicity = vasculopathy causing transient ischemic symptoms or stroke at a median 1.2 yrs (0.8–7.1) from completion of PBT. n = 6 (3.4%). Ten (6%) pts developed symptomatic brainstem toxicity, corresponding to a 3-yr actuarial rate of grade II brainstem toxicity of 5.5% (95% CI: 2.9–10.2). Median duration to toxicity onset: 3 months, & 9 of 10 toxicities occurred within 4 months. There were eight cases of grade II toxicity (4.5%), one case of grade III toxicity (0.5%), & one case (0.5%) of grade-5 brainstem toxicity. There were no radiation-induced second tumours or cases of cervical subluxation	See opposite
MacDonald (2013)[35]	Epend	Cervical subluxation (n = 2); post RT cavernomas (n = 2); necrosis (n = 1); no cases of secondary malignancies	NR
Sato (2017)[36]	Epend	Radiotherapy-related vasculopathy: n = 8/79 (10%) pts Radio-necrosis (n = 6): cranial nerve palsy: n = 1 (0.5 yrs post-XRT); dysarthria, somnolence & ataxia: n = 1 (0.33 yrs post-XRT); seizures: n = 1 (0.38 yrs post-XRT); acute right-side weakness: n = 1 (0.13 yrs post-PBT); progressive ataxia, cranial nerve palsies & weakness: n = 1 (0.33 yrs post-PBT); worsening balance, worsening VI nerve palsy & a speech problem (0.3 yrs post-PBT) in 1 patient who had posterior fossa syndrome & had residual cranial nerve palsy pre-PBT Stroke presented with acute onset of hemiparesis: n = 1 (1.7 yrs post-XRT) Cavernoma presented with seizure activity: n = 1 (4.2 yrs post-XRT)	1.6 – 4.2 yrs post-RT (see opposite for individual patient follow-up)
De Amorim Bernstein (2013)[37]	AT/RT	Febrile neutropenia^a^, bone marrow suppression^a^, mucositis^a^, cardiomyopathy^b^, hyponatremia^b^, nausea^c^, vomiting^c^, cranial nerve palsy^d^, focal weakness^d^ & seizures^d^ Most common AEs after RT were nausea/vomiting, note that all but 2 pts anaesthetized during RT	NR
Haskins (2015)[38]^e^	AT/RT	Based on 16 pts, skin erythema (grade I/II): n = 4 (25%); nausea & vomiting (grade II): n = 4 (25%); & weight loss & fatigue: n = 2 (13%) – likely to be related to chemo. No RT necrosis seen but ‘some’ cases of radiation associated change, therefore given a short course of steroids	NR
McGovern (2014)[39]	AT/RT	Grade I-II skin toxicities (erythema & alopecia): n = unspecified but occurred in ‘most pts’; Based on 27/31 pts who completed PBT, grade III-V^f^ acute toxicities reported during PBT included neutropenia (grade III): n = 2 (7%); thrombocytopenia (grade IV): n = 1 (4%); pancytopenia (grade IV): n = 2 (7%); emesis (grade III): n = 1 (4%); anaemia (grade III): n = 1 (4%); sepsis (grade IV): n = 1 (4%); hypertension (grade IV): n = 1 (4%); & death due to sepsis: n = 1 (4%) 5/31 pts also presented with clinical signs, substantiated by radiographic evidence, of radiation necrosis, including ataxia (grade II): n = 1 (20%), ataxia (grade III): n = 1 (20%); hypotonia (grade III): n = 1 (20%); quadriplegia (grade III): n = 1 (20%); bulbar palsies (grade IV): n = 2 (40%); & hemiparesis (grade III): n = 1 (20%). Pts with radiation necrosis treated with steroids and survived	Acute toxicities: during PBT Radiation necrosis toxicities: within 4 mths of completing PBT
Weber (2015)[40]^g^	AT/RT	Based on 15 (100%) pts, acute toxicities reported included bone marrow toxicities [grade I: n = 11 (73%); grade II: n = 2 (13%)]; alopecia: n = 15 (100%); & grade 1–2 erythema: n = 14 (93%). ‘Late toxicities’ included motor dysfunction [grade I: n = 1 (7%) & II: n = 1 (7%)]; one of these two pts experienced radiation necrosis, survived	NR
Bishop (2014)[42]	Cranio	Post-RT adverse events: vascular injuries (n = 5):- PBT: n = 2 (10%) versus XRT: n = 3 (10%); visual dysfunction (n = 5):- PBT: n = 1 (5%) versus XRT: n = 4 (13%); hypothalamic obesity (n = 13):- PBT: n = 4 (19%) vs IMRT: n = 9 (29%)	NR. Classified as ‘late morbidities ‘newly acquired from start of radiation’
Laffond (2012)[44]	Cranio	Based on 29 (100%) pts, epilepsy (n = 4; 14%); hemiparesis (n = 3; 10%); recurrent headaches (n = 15; 52%); visual impairment (reduced acuity and/or field loss) (n = 23; 79%); obesity [Body Mass Index > 97th percentile] (n = 17; 59%); & daily fatigue (n = 21; 74%)	NR
Luu (2006)[45]	Cranio	Based on 12 (80%) pts, stroke; n = 1 (8%) & posterior fossa meningioma: n = 1 (8%). – this pt received a previous course of external beam x-ray therapy as part of his initial treatment, developed a posterior fossa meningioma 59 months following salvage treatment with repeat resection & Adj PBT’	2.8 yrs post-primary treatment & 4.9 yrs post-salvage treatment respectively
Greenberger (2014)[47]	LGG	Visual acuity & optic nerve atrophy reduction: n = 3/18 (17%) high-risk pts who received a maximum RT dose to the optic chiasm, optic nerve or retina; Moyamoya disease: n = 2 pts with Neurofibromatosis type 1 (6%)	Visual acuity:’at most recent follow-up’; Moyamoya disease (n = 2): 1.0 & 0.9 yrs post-PBT respectively
Hug (2002)[48]	LGG	Otitis media (n = 1) requiring hospitalisation; Moyamoya disease (n = 1)	0.6—6.8 yrs post-completion of PBT
Indelicato (2019)[49]	LGG	*In pts free of tumour progression or pseudoprogression:-* •Significant permanent visual impairment due to retinopathy: n = 1 (0.6%)(optic pathway glioma) • Asymptomatic vasculopathy (grade 1 toxicity): n = 6 (3%), including cavernoma (n = 2) (1.1%), mild vessel stenosis (n = 3) (1.7%) or microcalcifications in the irradiated area (n = 1) (0.6%) *Serious RT-attributable late toxicity: n* = *7 (4%):-* •Second malignancy: n = 1 (a high-grade glioma in a 16-yr old 4 yrs after PBT for a grade 2 LGG) •Brainstem necrosis (requiring steroids): n = 2 pts with pilocytic astrocytoma at 6 & 11 yrs post-PBT •Vasculopathy: n = 3 (1.7%) •Visual decline: n = 1 following retinopathy impacting unilateral visuals field 2-yrs post-PBT, requiring laser ablation management	4.4 yrs
Farnia (2014)[51]	Pineo	Non-haematological acute toxicities:- local alopecia & mild-to-moderate nausea/vomiting: n = NR but ‘in almost all pts’ Haematological acute toxicities:- grade III neutropenia: n = 3^ h^; grade 3 anaemia: n = 1 Long term toxicities – (timescale NR) reported were: 2 pts with cognitive decline, 1 pt with grade III seizures, 1 pt with bilateral grade III avascular necrosis of the femoral head – all pts received photon RT	During PBT & long term

**Key:- a:** graded by Common Toxicity Criteria (version 3.0). According to the authors, ‘only acute toxic effects possibly, probably or definitely related to radiation were reported’; **b:** according to authors, these toxicities were ‘related to chemotherapy’; **c:** ‘adverse effects of irradiation’; **d:** ‘surgical complications’; **e:** according to the authors, while radiation necrosis did not occur in any of the patients, ‘radiation-associated change’ in some patients prompted short-term treatment with steroids; **f:** toxicities were graded in accordance with the Radiation Therapy Oncology Group (RTOG) Acute Radiation Morbidity Scoring Criteria; **g:** estimated 2-yr TFS for this study (n = 15) was 90% (CI95% 71.4–100); **h:** all three children underwent concurrent chemotherapy (i.e. vincristine alone or in combination with other agents).

A variety of late effects were reported. Endocrinopathies were reported in four studies (165 patients) [22, 24, 25, 31]. Yock reported at 3, 5 and 7 years post PBT, observing that deficiencies increased over time. By year 7, 61% (36/59) of patients had at least one endocrine deficiency, the most common being growth hormone deficiency (GHD) occurring in 31 patients [31]. Comparing PBT with photon RT, Eaton (2016) found a statistically significant reduction in the incidence of central hypothyroidism (p < 0.001) and sex hormone deficiency (p = 0.013) in PBT patients at 5.8 and 7-years follow-up [22] (Table 4).

**Table 4:** Summary of results of endocrinopathies in children and young adults treated with PBT for CNS tumours

Study details Author, year [ref]	Tumour type	N in study (assessed)	Any type of endocrinopathy (unspecified)	Central hypothyroidism	Growth hormone deficiency (GHD)	Thyroid deficiency	Adrenocortical insufficiency	Sex hormone abnormality
Eaton (2016)[22]	MB	77 (77) [PBT: n = 40^a^ XRT: n = 37]	Follow-up for all: PBT: 5.8 yrs (3.4–9.9) XRT: 7.0 yrs (3.5–13.5) Difference is statistically significant (P = 0.01)	PBT: n = 9 (23%) XRT: n = 26 (69%) (p < 0.001)	PBT: n = 21 (53%) XRT: n = 21 (57%) (p = 0.708); Need for endocrine replacement therapy:- PBT: n = 22 (55%); XRT: n = 29 (78%) (p = 0.047)	NR	PBT: n = 2 (5%) XRT: n = 3 (8%) (p = 0.667)	PBT: n = 1 (3%) XRT: n = 7 (19%) (p = 0.013) Precocious puberty:- PBT: n = 7 (18%) XRT: n = 6 (16%) (p = 0.881)
Grewal (2019)[24]	MB	14	NR	NR	NR	Hypothyroidism (grade 2) with growth delay n = 1	NR	NR
Jimenez (2013)[25]	MB (n = 12) sPNET (n = 3)	15 (13)	3.3 yrs (1.0–8.5 yrs): grade II endocrinopathies requiring HRT: 3/12 (25%)	NR	3.3 yrs (1.0–8.5): n = 1 (8%)^b^	3.3 yrs (1.0–8.5): n = 1 (8%)	3.3 yrs (1.0–8.5): n = 1 (8%)	3.3 yrs (1.0–8.5): premature puberty: n = 1 (8%)
Yock (2016)[31]	MB	59 (59)	All pts:- 3-yrs: 27% (95% CI: 16–39) 5-yrs: 55% (95% CI: 41–67) 7-yrs: 63% (95% CI: 48–75) Standard risk pts:- 3-yrs: 28% (95% CI:15–43) 5-yrs: 58% (95% CI:40–72) 7-yrs: 68% (95% CI:49–82 Intermediate-high risk pts:- 3-yrs: 25% (95% CI:9–46) 5-yrs: 50% (95% CI:26–70) 7-yrs: 50% (95% CI:26–70) P = 0.495	N	All pts:- 3 yrs: 22% (95% CI: 2–33) 5 yrs: 46% (95% CI:33–59) 7 yrs: 55% (95% CI:40–68) Standard risk pts:- 3 yrs: 23% (95% CI:11–37) 5 yrs: 50% (95% CI:33–65) 7 yrs: 62% (95% CI:42–76) Intermediate-high risk pts:- 3 yrs: 20% (95% CI:6–40) 5 yrs: 40% (95% CI:18–61) 7 yrs: 40% (95% CI:18–61) P = 0.368	All pts:- 3 yrs: 12% (95% CI: 5–22) 5 yrs: 21% (95% CI: 11–32) 7 yrs: 26% (95% CI: 15–38) Standard risk pts:- 3 yrs: 10% (95% CI: 3–22) 5 yrs: 21% (95% CI: 10–35) 7 yrs: 25% (95% CI: 12–40) Intermediate-high risk pts:- 3 yrs: 15% (95% CI: 4–34) 5 yrs: 20% (95% CI: 6–40) 7 yrs: 29% (95% CI: 9–53) P = 0.901	All pts:- 3 yrs: 5% (95% CI:1–13) 5 yrs: 9% (95% CI:3–17) 7 yrs: 9% (95% CI:3–17) Standard risk pts:- 3 yrs: 3% (95% CI: 0–12) 5 yrs: 3% (95% CI: 0–12) 7 yrs: 3% (95% CI: 0–12) Intermediate-high risk pts:- 3 yrs: 10% (95% CI: 2–28) 5 yrs: 20% (95% CI: 6–40) 7 yrs: 20% (95% CI: 6–40) P = 0.075	All pts:- 3 yrs: 3% (95% CI: 1–11) 5 yrs: 3% (95% CI: 1–11) 7 yrs: 3% (95% CI: 1–11) Standard risk pts:- 3 yrs: 3% (95% CI: 0–12) 5 yrs: 3% (95% CI: 0–12) 7 yrs: 3% (95% CI: 0–12) Intermediate-high risk pts:- 3 yrs: 5% (95% CI: 0–21) 5 yrs: 5% (95% CI: 0–21) 7 yrs: 5% (95% CI: 0–21) P = 0.638
Ares (2016)[32]	Epend	50 (50)	NR	3.6 yrs: permanent grade 2 requiring replacement: n = 3 (6%)	3.6 yrs: grade 2 requiring replacement: n = 3 (6%)	NR	NR	NR
Indelicato (2017)[34]	Epend	179 (179)	NR	NR	3.0 yrs: Hormone deficiency: n = 13 (7%), of which 11 had GHD (6%)	NR	NR	NR
MacDonald (2013)[35]	Epend	70 (32)	NR	3.5 yrs: n = 1/32 (3%)	3.5 yrs: n = 2/25 (8%) Deficient levels of IGF-1 (no diag of GHD): n = 9/25 (36%) (not on replacement therapy). 3.4yrs: Median change in height = median loss 2.6 percentiles (p = 0.14) 57pts	NR	NR	NR
De Amorim Bernstein (2013)[37]	AT/RT	10 (7)	NR	2.5 yrs (0.6–8.0): n = 2 (28%)	2.5 yrs (0.6–8.0): n = 3 (43%)^c^	NR	NR	NR
Bishop (2014)[42]^d,e^	Cranio	52 (52)	Panhypopituitarism (n = 24; 46%):- PBT: n = 7 (13%) XRT: n = 17 (33%) (p = 0.162); Other^f^ (n = 16; 31%):- PBT: n = 9 (17%) XRT: n = 7 (13%); (p = 0.139)	NR	NR	NR	NR	NR
Laffond (2012)[44]	Cranio	29 (29)	Hypothalamic syndrome: n = 18 (62%) Pituitary dysfunction: n = 28 (96%)	NR	NR	NR	NR	NR
Luu (2006)[45]	Cranio	16 (16)	3 yrs: panhypopituitarism n = 1 (6%)	NR	NR	NR	NR	NR
Jimenez (2021)[43]	Cranio	77(77)	Endocrinopathies Pre-RTPost-RT None 10 (13%)5 (6%) Any endocrinopathies 67 (87%)72 (94%) Panhypopituitarism 23 (30%)43 (56%) Other anterior hypopituitarism40 (52%)24 (31%) Diabetes insipidus 28 (36%)9 (12%) Pre- vs post-RT endocrinopathies Stable38 (49%) Worsened36 (47%) Improved3 (4%)					
Greenberger (2014)[47]	LGG	32 (29)	“N = 9 (31%) pts with intracranial tumours (31%) had 1 or more suspected neuroendocrine abnormalities resulting from tumour involving the HPA (hypothalamic pituitary adrenal axis) before they started RT, although definitive testing was often not performed until after RT. Endocrine abnormalities included growth hormone deficiency, cortisol insufficiency, testosterone deficiency, elevated prolactin, diabetes insipidus & precocious puberty (pt numbers for each endocrinopathy unspecified)^g,h^ All but one occurred in the high-risk dose group. Age (less than 8 yrs or less than 11yrs) was not found to have a statistically significant effect on endocrine outcomes					
Hug (2002)[48]	LGG	27 (27)	3.3 yrs: hypopituitarism: n = 4 (15%) – all had RT on tumours close to pituitary gland	NR	NR	NR	NR	NR
Indelicato (2019)[49]	LGG	174 (174)	Central hormone deficiency (new-onset, grade 2) post-PBT: n = 39 (22%):- Growth hormone deficiency: N = 31 (18%)	NR	Growth hormone deficiency: n = 31 (18%)	NR	NR	NR
MacDonald (2011)[50]	GCT	22 (22)	No pts developed diabetes insipidus	2.3 yrs (1.1–8.1): n = 2 (9%)	2.3 yrs (1.1–8.1): n = 2 (9%) Need for growth hormone replacement: N = 2 (9%)	NR	NR	NR

**Key:- a**: the 40 PBT in this case series study are a subset of the 59 PBT patients in the single arm phase II study by Yock 2016; **b**: at a median follow-up of 3.2 yrs (1.0–8.5) from completion of PBT, there was statistically significant reduction in age-adjusted height compared to baseline (median height percentile 25.79 and z score -0.65 (p = 0.03), although when the 3 patients with documented GHD were excluded from the analysis, the result was no longer statistically significant (p = 0.18); **c:** mean height z-score; a comparison of standard deviation of the patient height to the average height in the general population was 0.847 at baseline while at median follow up of 2.3 yrs (0.6 – 8.4) it was -0.735. At follow-up, only one patient had a z-score of less than -2; **d**: extent of surgery before RT did not correlate with post-operative endocrine or visual complications; **e**: endocrinopathies newly acquired from the start of RT; **f**: growth hormone deficits, hypothyroidism, adrenal insufficiency, sexual hormone deficiencies; **g**: all but one of the documented endocrinopathies (GHD, cortisol insufficiency, testosterone deficiency, elevated prolactin, diabetes and precocious puberty) occurred in the high-risk group (p < 0.0001). There was no significant effect on data dichotomised by age at time of PBT (less than 8 yrs versus more than 8 yrs) or the cohort’s median age of 11.0 yrs; h: these nine intracranial tumour patients were diagnosed post-PBT with endocrine deficiencies, although they were suspected of having endocrine abnormalities due to tumour activity prior to PBT

*AT/RT* Atypical Teratoid/Rhabdoid Tumour, *Cranio* Craniopharyngioma, *Diag* diagnosis, *Epend* Ependymoma, *GCT* Germ Cell Tumour, *LGG* low grade gliomas, *MB* medulloblastoma, *PBT* proton beam therapy, *Photon RT* photon radiotherapy, *(s)PNET* (supratentorial) Primitive Neuroectodermal Tumour, *NR* not reported

### Ependymoma

Conducted in three institutions, five case series studies (two prospective [32, 34] and three retrospective [33, 35, 36]) assessed the effects of PBT in 398 children with predominantly intracranial ependymoma. One study was comparative and compared PBT with patients who had received photon RT (non-randomised) [36]. The mean sample size was 80 and the median study follow-up was 3.6 years (Table 1).

Eighty-eight percent of patients were receiving first-line chemotherapy while 12% had recurrent local or metastatic disease [33–36]. Patients ranged from infants to young adults with median age within the studies ranging from 2.5 to 5.3 years. Patients received PBT as part of a multi-modal treatment regimen with patients undergoing surgical resection (78% achieving GTR) and chemotherapy (38%) prior to PBT/photon RT. The median dose of PBT was 55.8 Gy_RBE_ delivered in fractions of 1.8 Gy_RBE_ (Table 1 and SI Tale 1).

#### Tumour related outcomes

Survival was reported in all five studies. In patients treated with PBT, three-year OS ranged from 90% [34] to 97% [36] in patients receiving first-line therapy, with 3-year PFS ranging from 76% [34, 35] to 82% [36]. In Eaton’s study of 20 patients with recurrent disease, 3-year OS was 79% and PFS was 28% [33]. Comparing PBT with photon RT, Sato found statistically significant differences in favour of PBT for both 3-year PFS (82% versus 60%; p = 0.031) and local RFS (88% versus 65%; p = 0.01), but no statistical difference for OS [36]. Ares reported a 5-year OS of 84% in respect of 50 patients treated with pencil beam scanning PBT [32] (Table 2).

Failure rates were reported in all five studies. LFR at 3-years was 15% [34] and 17% [35] with 5-year LFR at 22% [32] and 23% [35]. DFR at 3-years was 15% [34] and 23% [35] and at 5-years 17% [35]. Median time to LFR and DFR was 1.4-years and 1-year, respectively [34]. In a univariate analysis LFR was related to extent of surgery (GTR: 21.6%, subtotal resection (STR): 35.5% (p = 0.003)) [34]. Comparing PBT with photon RT, Sato reported a LFR of 15% and DFR of 2% for PBT assessed at 2.6 years follow-up and LFR of 47% and DFR of 8% for photon RT assessed at 4.9 years follow-up, but this difference is likely to be due to the differences in follow-up times [36]. In recurrent patients 3-year LFR and DFR was 45% and 67%, respectively with second failure following first failure patterns [33] (Table 2).

#### Toxicity related outcomes

Short-term serious adverse events were reported in all five studies (398 patients) [32–36]. There were 14 cases of RT-associated vasculopathy presenting as stroke [34, 36] and radio-necrosis [36], 11 cases of brainstem toxicity including one fatality reported [32, 34, 36] as well as three cavernoma and two cervical subluxations [35] (Table 3).

Various medium-term and late endocrine toxicities were reported. Central hypothyroidism and GHD were the only endocrinopathies reported over three studies, with GHD being the most common [32, 34, 35] (Table 4.)

Ototoxicity was reported in three studies [32, 34, 35], but occurred at low levels and appeared to be related to prior cisplatin chemotherapy or in patients with the tumour close to the cochlea [32, 35] (Table 5).

**Table 5:** Summary of results of ototoxicity in children and young adults treated with PBT for CNS tumours

Study ID Author, year [ref]	Tumour type	N in study (assessed)	Details of ototoxicity
Grewal (2019)[24]	MB	14	Grade II bilateral hearing loss n = 2 (both received Cisplatin)
Jimenez (2013)[25]	MB	15 (13)	Grade III-IV ototoxicity: 3.2 yrs: n = 2 (24%) Hearing amplification: 3.2 yrs: n = 6 (46%) including bilateral, FM amplifier: n = 3 (23%); bilateral, hearing aids: n = 3 (23%) High-frequency SNHL: 3.2 yrs (1.0–6.7): n = 9 (70%):- bilateral: n = 8 (62%); right: n = 1 (8%) (of these 5 pts had bilateral sensorineural hearing loss before the initiation of PBT)
Moeller (2011)[27])	MB	23 (19) (35 ears)	Grade III-IV ototoxicity: 1- yr: n = 1 (5%) Hearing amplification: 1-yr: n = 3 (16%) NB: Scatter plot analysis revealed ‘no obvious correlation’ between RT dose to the cochlear and ototoxicity
Paulino (2018)[28]	MB	84 (84):- PBT: 38 (75 ears) XRT: 46 (91 ears)	Grade III-IV ototoxicity: PBT:- 4.7 yrs: SIOP Boston^a^: 20%; Brock^b^: 9%; POG^c^: 17%; CTCAE: 30% XRT:- 5.5 yrs: SIOP Boston: 23%; Brock: 10%; POG: 21% CTCAE: 28% POG hearing score- % of ears with each score (PBT vs XRT):- Score 0: 37 vs 26; Score 1: 31 vs 44; Score 2: 15 vs 9; Score 3: 13 vs 19; Score 4: 4 vs 2 SIOP Boston hearing score—% of ears with each score (PBT vs XRT):- Score 0: 37 vs 33; Score 1: 28 vs 36; Score 2: 15 vs 8; Score 3: 15 vs 16; Score 4: 5 vs 7 Brock hearing score—% of ears with each score (PBT vs XRT):- Score 0: 35 vs 32; Score 1: 32 vs 38; Score 2: 24 vs 20; Score 3: 5 vs 7; Score 4: 4 vs 3
Yock (2016)[31]	MB	59 (49) (98 ears)	Grade III-IV ototoxicity: 3-yr (n = 45): 12% (95% CI: 4–31) (NB: these 45 pts had no grade 3/4 hearing loss at baseline) 5-yr: 16% (95% CI: 6–29) POG hearing score: 5 yrs: Same/improved by 1 point: 34 ears (35%); Worsened by:- 1 point: n = 21 ears (21%); 2 points: n = 35 ears (36%); 3 points: n = 6 ears (6%); 4-points: n = 2 ears (2%) ‘Overall, hearing loss was statistically significantly worse at follow-up compared to baseline (p < 0.0001). Excluding pts with grade 3–4 hearing loss at baseline, 10/90 (11%) ears developed grade 3–4 hearing loss in both ears & 2 (4%) developed it in 1 ear’. ototoxicity was not significantly associated with sex, age, shunt placement, cumulative cisplatin dose, or mean dose to cochlea’.’
Ares (2016)[32]	Epend	50 (50)	Defined as ‘late toxicity – 90 days post PBT Grade I unilateral hearing loss n = 1 (2%) Grade III-IV ototoxicity: n = 2 (4%) definitive unilateral deafness (both pts with infratentorial tumours infiltrating into the internal acoustic canal, received PBT to ipsilateral cochlea)
Indelicato (2017)[34]	Epend	179 (179)	Hearing amplification: 3.2 yrs: new hearing loss requiring hearing aids: n = 11 (6%); 7 bilateral & 4 unilateral deficits. Note: of these 8/11 received cisplatin including 6/7 with bilateral hearing deficits
MacDonald (2013)[35]	Epend	70 (23)	Hearing loss (grade not specified): 2.3 yrs: n = 2 (9%) with infratentorial tumours, who received higher RT to cochlea due to tumour extension into the foramen of Luschka
De Amorim Bernstein (2013)[37]	AT/RT	10 (10)	High-frequency SNHL: 2.3 yrs (0.9–8.3): n = 1 (10%) – developed after cisplatin chemo
Haskins (2015)[38]	AT/RT	16	1 = difficulty hearing due to cochlear damage, tumour next to cochlea
Bass (2018)[41]	CRANIO	74 (74)	SNHL:- Clinically significant SNHL assessed according to Chang Ototoxicity Grading Scale^d^: At most recent evaluation compared to baseline, 0 pts had SNHL in the Conventional Frequency (CF) range (0.25 – 8.0 kHz) while 2 pts (3%) had SNHL (Chang Grade 1a) in the Extended High Frequency (EHF) range (9.0 – 16.0 kHz): - 1 pt received 0.3 & 6.6 Gy (RBE) to the right & left ears, respectively, & had left ear Chang Grade 1a at frequencies ≥ 10 kHz ranging in severity from moderate to moderately severe; - 1 pt received 25.8 & 54.2 Gy (RBE) to the right & left ears, respectively, & had bilateral Chang Grade 1a at frequencies ≥ 10 kHz for the right ear & ≥ 9 kHz for the left ear that fell within the moderate severity range Non-clinically significant SNHL assessed according to the ASHA^e^ criteria: At last evaluation compared with baseline measures, a decrease in hearing was observed in 0 pts in the CF range alone, in 9 pts (12%) in the EHF range alone, & in 15 pts (20%) in both the CF & EHF ranges Distorted Product Otoacoustic Emissions (DPOAEs)^f^: Ototoxic DPOAE levels (defined as a decrease of ≥ 6 dB at one or more *f*2 frequencies) were greater at higher compared with lower frequencies for both left & right ears; for example, based on the number of eligible left ears [which ranged from 60 (at 1.5 kHz) to 31 (at 8 kHz)], ototoxic DPOAE levels ranged from 18% at 1.5 kHz to 45% at 8 kHz Speech-in-Noise (SIN)^g^: For 41 evaluable pts, there was no decline in SIN perception from baseline to last evaluation (p = 0.6463)
Indelicato (2019)[49]	LGG	174 (174)	4.4 yrs: Grade II: partial in 1 ear post-PBT: n = 4 (2%) Grade III-IV with need for amplification: Grade III: n = 1 (0.6%)
Farnia (2014)[51]	PINEO	22 (22)	Grade III: n = 1 (4%) – pt received photon RT

**Key:- a:** SIOP Boston ototoxicity grading scale:- Grade 3: > 20 dB loss at ≥ 2 kHz; Grade 4: > 40 dB loss at ≥ 2 kHz; **b:** Brock ototoxicity grading scale:- Grade 3: ≥ 40 dB loss at ≥ 2 kHz; Grade 4: ≥ 40 dB loss at ≥ 1 kHz; **c:** Pediatric Oncology Group (POG) ototoxicity grading scale:- Grade 3: > 40 dB loss at > 2 kHz; Grade 4: 40 dB loss at < 2 kHz; **d:** the Chang Ototoxicity Grading Scale assesses clinically significant SNHL and utilises absolute hearing threshold levels highly correlated with recommendations for audiologic intervention. Grade 0 (no complications): 20 dB at 1, 2, and 4 kHz; grade 1a: ≥ 40 dB at 6–12 kHz; grade 1b: > 20 and < 40 dB at 4 kHz; grade 2a: ≥ 40 dB at ≥ 4 kHz; grade 2b: > 20 and < 40 dB at < 4 kHz; grade 3: ≥ 40 dB at ≥ 2 kHz; grade 4 (severe complications): ≥ 40 dB at ≥ 1 kHz. Although initially developed to assess clinically significant platinum-induced ototoxicity, the Chang Ototoxicity Grading Scale has been used to rate radiation-induced ototoxicity, particularly because it emphasizes SNHL in the higher frequencies which are more severely affected by RT) and it includes a criterion (Grade 2b) that captures milder degrees (low or mid-frequency) RT-induced SNHL (Bass et al., 2016); **e:** The American Speech-Language-Hearing Association (ASHA) criteria identify a change (i.e., decrease) in hearing sensitivity when compared to baseline measures as follows: (a) ≥ 20 dB HL decrease in pure-tone threshold at a single test frequency, (b) ≥ 10 dB HL decrease in threshold at two adjacent frequencies, or (c) loss of response at three consecutive frequencies where responses were previously obtained. The ASHA criteria are used as a binary outcome (yes or no) measure designed to detect early ototoxic changes before clinical SNHL occurs; **f:** otoacoustic emissions (OAEs) are sounds measured in the external ear canal that reflect movement of the outer hair cells in the cochlea. Normal outer hair activity is essential for auditory function, and significant decreases in OAEs provide early and strong evidence of hearing dysfunction; **g:** speech-in-noise (SIN) testing is used to assess the functional impact of ototoxicity by evaluating the patient’s ability to comprehend speech (i.e., monosyllabic words or sentences) in the presence of background noise

*AT/RT* Atypical Teratoid/Rhabdoid Tumour, *CTCAE* Common Terminology Criteria for Adverse Events, *Epend* Ependymoma, *MB* Medulloblastoma, *PBT* proton beam therapy, *Photon RT* photon radiotherapy, *PIN* Pineoblastoma, *POG* Pediatric Oncology Group, *pt* patient, *SIOP* International Society of Pediatric Oncology, *SNHL* Sensorineural Hearing Loss, *Yrs* years

Neuro-cognitive outcomes were only assessed by MacDonald (2013) who reported small and non-statistically significant increases in both mean Full Scale Intelligence Quotient test (FSIQ) (n = 14) and adaptive skills/functional independence (n = 28) at 2.2 years follow-up compared to baseline [35] (Table 6).

**Table 6:** Summary of results of neuro-cognitive and adaptive behaviour outcomes

Study ID Author, year [ref]	Tumour type	Study N (assessed)	Baseline & follow-up assessment times (range)	Mean Full Scale Intelligence Quotient (FSIQ)	Mean Verbal Comprehension Index (VCI)	Perceptual Reasoning Index (PRI)	Working memory	Processing speed	Scales of Independent Behaviour Revised (SIB-R)
Jimenez (2013)[25]	MB/ sPNET	15 (5–8)	Baseline: start of PBT Follow-up (median): 2.2 yrs (1.3 – 3.2)	Based on n = 5: Baseline: mean 108.2; Follow-up: mean 105.8 (p-value: NR^a^)^b^	NR	NR	NR	NR	Based on n = 8: Baseline: 106.3; Follow-up: 116.3 (p-value: NR^a^)^c^
Kahalley (2019)[21]	MB	79 (70)	Baseline timing: NR Follow-up: yearly Mean follow-up: 4.3 yrs	Measured global IQ PBT: stable overtime, RT significant decline = 0.9 points per yr	No significant differences between the two groups	PBT: mean change per yr = 1.0 point (P = 0.053) RT: mean change per yr: 0.8 (P = 0.206)	Based on n = 70 PBT: stable working memory (P = 0.891) RT: mean change -2.2 per yr (P = 0.001)	Both groups had a decline with an average of 0.90 points per yr observed	NR
Yock (2016)[31]	MB	59 (49–54)	Baseline: within two weeks of start of PBT Follow-up (median): 5.2 yrs (2.6–6.4)	Based on n = 54 Baseline: 104.5 (95% CI: 101.3 to 107.7) Mean change per yr: -1.5 (95% CI: -2.1 to -0.9) (p < 0·0001)^d,i^	Based on n = 53 Baseline: 109.2 (95% CI: 106% to 112.4%) Mean change per yr: -1.3 (95% CI: -2.0 to -0.7%) (p < 0.0001)^e,j^	Based on n = 53 Baseline: 103.5 (95% CI: 100.2 to 106.8) Mean change per yr: -0.4 (95% CI: -1.0 to 0.3) (p = 0.25)^f,j^	Based on n = 41 Baseline: 98.7 (95% CI: 94.0 to 103.3); Mean change per yr: -0.8 (95% CI: -1.8 to 0.3) (p = 0.17)^g,j^	Based on n = 49 Baseline: 95.3 (95% CI: 91.5 to 99.2); Mean change per yr: -2.4 (95%CI: -3.2 to -1.6) (p < 0·0001)^h,j^	NR
MacDonald (2013)[35]	Epend	70 (14–28)	Baseline: start of PBT Follow-up (mean):- FSIQ: 2.1 yrs (1.0–4.5) SIB-R: 2.2 yrs (1.0–5.9)	Based on n = 14:- Baseline: 108.5 Follow-up: 111.3 (p = 0.475)^k^	NR	NR	NR	NR	Based on n = 28:- Baseline: 100.1 Follow-up: 100.8 (p = 0.809)^l^
Jimenez (2021)[43]	Cranio	65/77 (84%) had at least 1 baseline test FSIQ n = 25pts: processing speed index (n = 20pts) Immediate & delayed verbal & visual memory n = 14pts; Scales of independent behaviour revised n = 30pts	41/65 (63%) had 6 mths fu	Stable	Stable	NR	Stable	Stable	Statistically significant decrease P = 0.011) in mean score at follow-up compared with the baseline. Not considered clinically significant as it was < 15 points change in overall score
Greenberger (2014)[47]^m^	LGG	32 (11–12)	Baseline: start of PBT Follow-up (mean):- FSIQ: 4.5 yrs (1.2–8.1) VCI: 4.9 yrs (1.2–8.1) PRI: 4.9 yrs (1.2–8.1)	Based on n = 11:- Baseline: 109.3 (SD 9.3); follow-up: 108.5 (SD 12.3); Mean change: -0.7 (SD 9.2); (p = 0.8)^n^ High-risk dose n = 4 Baseline: 107.3 (SD7.8); follow-up: 97 (9.7); Mean change: -10.3 (2.5); (p0.0038)	Based on n = 12:- Baseline: 113.2 (SD 12.9); follow-up: 112.7 (SD 13.9); Mean change: -0.5 (SD 11.7); (p = 0.88)^n^ High-risk dose n = 4 Baseline: 117.8 (SD7.8); follow-up: 104.3 (17.8); Mean change: -13.5 (3.3); (p0.0039)	Based on n = 12:- Baseline: 107.7 (SD 10.5); follow-up: 107.5 (SD 13.2); Mean change: -0.17 (SD 9.8); (p = 0.95)^n^	NR	NR	NR

**Key:- a:** no p-value reported but the authors stated that there were ‘no significant differences between baseline and follow-up mean Intelligence Quotient (IQ) scores or baseline and follow-up SIB-R scores.” **b:** IQ was assessed using one of the following age-appropriate measures: Bayley Scales of Infant Development (2nd edition), the Wechsler Preschool and Primary Scales of Intelligence (3rd edition) and the Wechsler Intelligence Scale for Children (4th edition) [All mean: 100; standard deviation: 15]; **c:** Age-appropriate behaviour and functional Behaviour assessed using the Scales of Independent Behavior, Revised (SIB-R) [Mean: 100; standard deviation: 15], a standardised questionnaire designed to assess four areas of adaptive behaviour: motor skills, personal living, social interaction and communication, and community living; **d:** FSIQ assessed using Wechsler Preschool & Primary Scales of Intelligence‐3rd Ed. (WPPSI‐III) (2.5–5 yrs), Wechsler Intelligence Scale for Children –4th Ed. (WISC‐IV) (6‐15 yrs) & Wechsler Adult Intelligence Scale‐3rd or 4th Edition (WAIS III or IV) (16 + yrs); **e:** VCI assessed using either Expressive Vocabulary Test‐2nd Ed or Peabody Picture Vocabulary Test‐4th Ed. (PPVT‐4) (2.5 yrs +) or Expressive One‐Word Vocabulary Test (2.5–25 yrs); **f:** PRI assessed using Beery‐Buktenica Developmental Test of Visual‐Motor Integration Version 5 or 6 (3 + yrs), Grooved Pegboard Test (5 + yrs) and Purdue Pegboard Test (4‐15 yrs); g: Working memory assessed using Children's Memory Scale ‐ Stories I & II (6‐15 yrs) or Wechsler Memory Scale‐3: Family Pictures and Logical Memory (16 + yrs); **h:** Processing speed assessed using Wechsler Intelligence Scale for Children‐ 4th Ed, Subtests: Coding & Symbol Search (6–15 yrs) or Wechsler Adult Intelligence Scale‐(WAIS‐III or WAIS‐IV), Subtests: Digit Symbol‐Coding and Symbol Search (> 16 yrs); **i:** No statistically significant differences in mean change per year in scores between standard risk patients & intermediate-high risk patients, male versus female, age < 8 yrs versus ≥ 8 years, CSI dose (18–27 Gy_RBE_ versus 36 Gy_RBE_); the only significant difference observed was between patients who had a involved boost field only versus those who had a whole posterior fossa boost. However, this was potentially confounded by differences in age with median age in the involved field group: 5.5-years (IQR: 4.0–8.5) versus 7.0-years (IQR: 5.0–10.0) in the posterior fossa group; j: no statistically significant differences in mean change per year in scores between standard risk patients & intermediate-high risk patients; k: FSIQ assessed using Bayley Scales of Infant Development or Weschler Preschool and Infant Development test or Weschler Intelligence scale for children; **l:** Adaptive skills and functional independence were assessed using the Scales of Independent Behaviour–Revised (SIB-R) reference (mean = 100; SD = 15) completed by parents; **m:** Across the strata of tumour location (supratentorial versus infratentorial), PBT dose and RT at less or more than 7-years of age; only results on FSIQ & VCI showed a significant decrease for patients treated with high-risk dose (20% volume dose to the left temporal lobe or hippocampus of ≥ 15 GYRBE) on FSIQ (n = 4) and began RT at < 7-year and high-risk dose (both n = 4) for VCI. Distinguishing the effects of age and dose for changes in VSI confounded by 3 out of 4 of the age < 7-years group also being classified as within the high-risk dosage group; n: assessed using Wechsler Intelligence Scale for Children-IV (WISC-IV) for a subset of patients with intracranial tumours

*Epend* Ependymoma, *LGG* Low grade gliomas, *MB* Medulloblastoma, *NR* not reported, *(s)PNET* supratentorial Primitive Neuroectodermal Tumour, *SD* standard deviation

No studies reported quality of life measures.

### Atypical teratoid/rhabdoid tumours (AT/RT)

Conducted in separate institutions, four single-arm, retrospective case series studies assessed PBT in 72 children with AT/RT [37–40]. The mean sample size was 18 and study follow-up ranged from 2.0 to 3.2 years.

All patients were receiving first-line therapy and 28% had confirmed metastatic disease at presentation. Mean age across the studies was 1.7 years. Prior to PBT, 97% of patients underwent surgical resection (47% achieved GTR) followed by induction chemotherapy (92%). The average PBT dose was 50.4 Gy_RBE_ in two studies [37, 39] and 54 Gy_RBE_ in two studies [38, 40] delivered in fractions of 1.8 Gy_RBE_. Chemotherapy was delivered either concurrently (25%) or post-PBT (67%) (Table 1 and SI Table).

#### Toxicity related outcomes

All four studies reported comprehensive lists of adverse events. Radiation necrosis was reported in six patients all of whom survived [38, 40] (Table 3).

Endocrinopathies and ototoxicity were assessed by De Amorim Bernstein in seven (70%) and ten patients, respectively (100%). Two patients (28%) developed hypothyroidism and three (43%) GHD at 2.5 years. One patient developed high-frequency sensorineural hearing loss (SNHL) at 2.3 years follow-up [37] (Tables 4 and 5).

HRQoL was assessed by Weber in 15 children, predominantly less than 2 years of age. Based on parental proxy reports, there was little variation between mean scores for physical, social, emotional and psycho-social functioning at two-months follow-up compared with baseline [40] (SI Table 2).

#### Tumour related outcomes

Survival was reported in all four studies with variable follow-up schedules possibly impacting estimates. OS ranged from 53% at 2 years [39] to 90% at 2.3 years [37]. PFS ranged from 46% at 2 years [39] to 75% at 1.4 years [38] (Table 2).

Failure rates were reported in three studies (n = 41). LFR ranged from 0 to 20%, and DFR 20% to 27% [37, 38, 40] (Table 2).

### Craniopharyngioma

Six studies assessed the effects of PBT in 272 children with craniopharyngioma. Of these, five were single arm retrospective case series [41, 43–46] and one was an historical control study, comparing PBT with photon RT [42]. The average sample size was 45 and study follow-up ranged from 2.0 to 6.2 years (Table 1).

Fifty-one percent of patients were receiving first-line therapy and 49% had recurrent disease [42–46]. Patient age ranged from 1.3 to 20 years [43–46]. Prior to radiotherapy, 97% of patients underwent surgical resection (69% STR, 11% GTR) and 20% either had a cyst drainage, fenestration or shunt inserted [41–44, 46]. The median dose of PBT ranged from 50.4 to 59.4 Gy_RBE_ delivered in fractions of 1.8 Gy_RBE_ (Table 1 and SI Table 1).

#### Tumour related outcomes

OS was reported in three studies (n = 149) [42, 43, 45]. Comparing PBT and photon RT, Bishop reported a non-statistically significant difference in 3-year OS between 21 patients who received PBT (OS 94%) and 31 patients who received photon RT (OS 97%) [42]. In 77 patients treated with PBT, 5-year OS was 97.7% [43]. Luu (n = 16) also reported a 5-year OS of 100% for patients who had undergone one surgical resection compared to 60% for those with more than one resection [45]. PFS was not reported (Table 2).

Specific to craniopharyngioma, Bishop reported NFFS and CFFS. No statistically significant differences were found in 3-year NFFS (92% versus 96%; p = 0.54) or 3-year CFFS (67% versus 77%; p = 0.99) between the PBT and photon RT groups [42].

LFR was reported in three studies. Winkfield (n = 24) reported LFR at 0% at 3.4 years [46]. In Luu (n = 16) and Jiminez (n = 77) the 5-year LFR was 6% and 10%, respectively [43, 45]. Median time to failure from PBT completion was 3.6 years (range 1.8–8.4) (Table 2).

#### Toxicity related outcomes

Bishop reported no significant differences in the incidence of post-RT vasculopathy, visual dysfunction and obesity between PBT and photon RT [42] (Table 4 and 5). In the Jiminez report one patient had vasculopathy symptoms (1.3%), one patient had a stroke (1.3%) and one Moyamoya syndrome (1.3%). Jiminez also reported visual outcomes including pre and post PBT, with 68% experiencing stable vision, 10% worsening, 10% improving and 12% unknown [43] (Table 3).

Endocrinopathies were reported in four studies [42–45]. Bishop reported no statistically significant difference between PBT and photon RT patients in the incidence of endocrinopathies newly acquired from the start of RT. The most common endocrinopathy was panhypopituitarism occurring in seven (13%) PBT and 17 (33%) photon RT patients (p = 0.162) [42]. Luu reported just one patient (6%) with panhypopituitarism [45], while Laffond reported pituitary dysfunction in 28 patients (96%) and hypothalamic syndrome in 18 PBT patients (62%) between 1.7 and 14 years follow-up [44]. Jiminez measured endocrinopathies pre- and post-PBT and found 49% were stable, 47% worsened and 4% improved [43] (Table 4).

Ototoxicity was comprehensively reported by Bass. Rates were low for clinically significant SNHL in the extended high frequency (EHF) range at 3% [41] (Table 5).

Neurocognitive outcomes were reported by Jiminez [43]. FSIQ, verbal and visual memory scores were stable, with adaptive skills (Scales of Independent Behaviour Revised (SIB-R)) had a statistically significant decrease in mean follow-up score compared with baseline, however this was not considered clinically important (Table 6).

HRQoL and executive functioning outcomes were reported by Lafford [44]. HRQoL was assessed via patient and parental proxy reported scores in 22 PBT patients (nine of which also received photon RT). At 3.4 year follow-up, overall HRQoL was deemed satisfactory, although between 25 and 50% of scores were indicative of low HRQoL for seven of the ten sub-domains. Fifty percent of patients had mild-moderate mood disorders, but no patients experienced severe depression. With respect to executive function, 24–38% of patients experienced problems with flexible thinking (‘shift’), emotional control and working memory (SI Table 2).

### Low grade glioma (LGG)

Three non-comparative single centre case series studies (one prospective [49] and two retrospective [47, 48]) assessed the effects of PBT in 233 children with LGG. The two retrospective studies had small sample sizes and both started recruitment in the 1990s, however, the prospective study by Indelicato involved 174 patients and was conducted between 2007 and 2017. Study follow-up ranged from 3.3 to 7.6 years.

Reported in two studies (n = 59), 75% were newly diagnosed while 25% had recurrent disease [47, 48]. No patients had metastatic disease. Mean patient age at time of PBT ranged from 8.7 to 11 years, although most included children from 2 to 21 years. Prior to PBT, a selection of patients underwent surgery (87%) followed by chemotherapy (44%) [47, 49]. One-hundred and seventy patients in the Indelicato series had > 0.5 cm gross disease at time of irradiation, the remaining four patients received RT due to multiple prior recurrences [49]. The average dose of PBT was 54 Gy_RBE_ (Table 1 and SI Table 1).

#### Tumour related outcomes

Survival was reported in all three studies. OS rates of 85%, 92% and 100% were reported at 3.3, 5.0 and 8.0 years follow-up, respectively [47–49]. PFS, reported in two studies (n = 206) was 84% and 90% at 5.0- and 6.0 years, respectively [47, 49] (Table 2).

LFR, reported in two studies, was 22% and 15% at 3.3 and 5.0 years, respectively [48, 49]. DFR reported in one study was 0% at 3.3 years [48] (Table 2).

#### Toxicity related outcomes

Indelicato reported serious PBT-attributable late toxicities in seven patients (4%), most notably brainstem necrosis (treated with steroids), vasculopathy and second malignancy [49] (Table 3).

Across the studies, endocrine abnormalities were reported in 23% of patients assessed, including hypopituitarism [48], growth hormone deficiency [49] and cortisol insufficiency [47] (Table 4).

Reported in one study, there was no significant decline in neuro-cognitive outcomes (FSIQ, verbal comprehension or perceptual reasoning) at 5-years relative to baseline in 12 patients (38%) assessed [47]. Visual acuity, assessed in 18 patients, was stable/improved relative to baseline in the 15 non-high-risk patients [47]. Ototoxicity was assessed in 174 patients, at 4.4 years, 4 patients (2%) had grade II partial hearing loss in one ear and one patient had grade III hearing loss with need for amplification [49] (Table 5 and 6).

For HRQoL, Hug reported that of 27 patients, no patient experienced a drop of more than 10% in the Lanksky performance scale [48] (SI Table 2).

### Germ cell tumours (GCT)

One single-arm retrospective case series by MacDonald, reported the effects of PBT in 22 children (mean age 11 years) with newly diagnosed GCT [50]. Fifty-nine percent had germinoma and 41% non-germinomatous germ-cell tumours (NGGCT) (Table 1 and 2). OS and PFS were 100% and 95%, respectively at 2.3 years follow-up. No patients experienced a local failure whilst DFR rates were 0% and 11% for germinoma and NGGCT patients, respectively (Table 2). Two patients (9%) experienced hypothyroidism and two (9%) required growth hormone replacement at 2.3 years. No patients developed RT-related diabetes insipidus (Table 4).

### Pineoblastoma

One study by Farnia reported the effects of PBT in children with pineoblastoma [51]. Undertaken in a single institution between 1982 and 2012, this historical control study included 22 patients under 25 years, of which 11 received PBT and 11 received photon RT and one gamma knife treatment. Median age was 7.7 years and 14.5 years for PBT and photon RT, respectively (Table 1). Survival and recurrence rates between PBT and photon RT were not statistically different (Table 2). Long-term toxicities—which all occurred in patients treated with photon RT—included grade 3 cognitive decline (n = 3), grade 3 seizures (n = 1), grade 3 hearing impairment (n = 1) and grade 3 avascular necrosis of the femoral head (n = 1) (Table 3, 5 and 6).

## Discussion

The aim of this systematic review was to investigate if the published clinical evidence supports the assumptions derived from dosimetry studies of PBT compared with photon RT in terms of equivalent survival, improved quality of life and/or reduced long-term treatment sequelae. Furthermore, recommendations for improving the quality and consistency of output data are presented.

In order to minimise bias we have undertaken this systematic review according to Cochrane methodology, which is designed to produce a systematic review that is as free as possible from methodological flaws, is reproducible and transparent. Our scoping search identified three previous systematic reviews, however, all are out of date with searches up to 2014 [11–13]. The review by Laprie 2015 [11] was the most closely aligned to our review, with aims to examine PBT and photon RT in children with brain tumours. However, some of the methodology that they have used may have introduced bias, for example they only utilised the database Medline, only sought English language publications, did not have an a priori protocol, did not quality assess the included studies and their searches were up to 2014. Systematic review is a powerful tool, but is by nature a retrospective exercise and governed by the available evidence. In rapidly evolving fields such as PBT it is important that reviews are regularly updated to ensure that they include all of the evidence and are as up-to-date as possible.

Thirty-one full-text published studies involving 1,730 children met our inclusion criteria. All but five studies [21, 32, 40, 41, 44] were conducted in the USA. Publication dates ranged from 2002 [48] to 2021 [43]. Studies were undertaken from 1982 [51] to 2018 [21]. Most of the patients were treated between the years 2000 and 2015, so the studies in this review are fairly similar regarding the dates, therefore any era differences may be small within this data set. There was one phase II single-arm study, six prospective case series studies, with one of these being comparative and 24 retrospective studies with seven of these being comparative. No RCTs were identified. Largely because of referral patterns in the USA, all the case series used opportunity sampling, i.e. data was based on patients referred to the proton centre routinely, not part of a specific PBT clinical trial, and in terms of the retrospective studies this was derived mainly from patient records. Tumour types included: medulloblastoma (11 studies); ependymoma (5 studies); ATRT (4 studies); craniopharyngioma (6 studies); LGG (3 studies); GCT (1 study) and pineoblastoma (1 study).

The studies were heterogeneous regarding aims and objectives, patient diagnoses, patient populations (some assessed younger patients) and outcomes. For this review we identified nine outcomes of interest. Five measured disease control (OS, PFS/RFS, LFR DFR), four measured treatment related short- to long-term side effects (adverse events, endocrinopathy, ototoxicity, neurotoxicity), and one measured treatment related HRQoL. Across the studies OS was the most frequently reported outcome, followed by LFR, and endocrinopathy. Adverse event reporting was inconsistent across the tumour types making it impossible to assess the incidence across the dataset. However, there were some serious adverse events reported—albeit in very small numbers—such as radio-necrosis, stroke and brainstem toxicity [24, 31–36, 38, 40, 45, 49]. Outcomes least reported were HRQoL, neurocognitive and ototoxicity. HRQoL was reported in just three tumour types (medulloblastoma, AT/RT, craniopharyngioma) and neurotoxicity in four tumour types (medulloblastoma, ependymoma, craniopharyngioma, LGG). Given that a reduction of late effects is the proposed key advantage of using PBT, it is disappointing that few studies reported these outcomes. Some study authors commented on the difficulty in obtaining long-term follow-up data as many patients had travelled from other hospital facilities to receive PBT and long-term outcomes were either not evaluated at or not reported to the proton centres. The difficulty in acquiring long-term late effects and HRQoL data has been an issue for many paediatric cancer trials including those which have included RT delay or avoidance. Prospective initiatives such as the USA Pediatric Proton Consortium Registry may yield more useful data in the future [52, 53] but may not be able to solve all these problems [54].

Ependymoma provided the most comprehensive dataset, both in terms of the number of outcomes measured and the proportion of patients in each study evaluated per outcome. The remaining tumour types were either inconsistent in terms of outcomes reported, only included a small percentage of the available patients across the outcomes or as in the case of GCT, pineoblastoma and AT/RT, were extremely limited in the number of patients available, therefore caution must be used in interpreting the results due to lack of power of the dataset.

OS was the most common outcome measure. Generally, for standard paediatric CNS indications, the rates of tumour control and hence cure are expected to be the same for protons as for photons. Most of the patients included in this review were newly diagnosed. OS was reported to be 100% to 68% depending on patient characteristics, follow-up times, etc. however without a randomised comparator it is not possible to “prove” whether PBT offers better, worse or equivalent disease control compared to photon RT. On the other hand, conducting survival equivalence randomised trials in a variety of different histological types with small patient numbers is probably not achievable. Taking into account the totality of radiobiological data and clinical experience it is universally accepted that considering the RBE of PBT tumour control and hence OS are equivalent.

Our systematic review included eight comparative studies, but these utilised either historical [28, 30, 36, 42, 51] or opportunity controls [21–23]. The main problem with the use of historical controls is confounding due to temporal shifts in care [55], particularly in older historical controls [28, 42, 51]. This is particularly pertinent to radiotherapy practices which has seen a shift from whole brain radiotherapy to more localised treatments, which may have impacted long-term adverse events and HRQoL. In addition, the multimodality of brain tumour treatment and improvements in delivering photon RT may also have had a substantial impact on disease control in historical comparisons. Temporal shifts may also have improved the accuracy of outcome assessment measures, for example, improvements in imaging may make adverse events such as radio-necrosis easier to identify and appear more common in newer studies, a consideration when comparing PBT radio-necrosis event rates with those from historical controls treated with photon RT. In studies using opportunity controls, the main problem is selection bias where patients not receiving PBT may not have been eligible to receive it and are therefore fundamentally different in terms of prognosis. This is exemplified by Sato, where 93% of patients receiving PBT had had a GTR at surgery compared to 76% of photon RT patients, indicating patients given photon RT were in the higher risk group, potentially biasing survival outcomes in favour of PBT [36].

Retrospective opportunity sampling also limits the type and methods of data collection. Across the studies, measurement and reporting of outcomes (particularly in patients with the same tumour type) were inconsistent, making between study comparisons difficult. One study which reported outcomes measured from diagnosis and completion of PBT demonstrated a marked difference between the two time points, with 2-year OS at 68% when measured from diagnosis and 48% when measured from PBT—a difference of 20% [39]. By using prospective data collection researchers can control what data are collected and the methods of collection. Utilising data from clinical trials investigating non-radiotherapy questions, such as the ongoing SIOP (International Society of Paediatric Oncology) Ependymoma II study [56] and the PNET5 study [57] which include patients treated with both PBT and photon RT can allow better prospective control on data collection. Although non-randomised, data derived from prospective trials also provides data with associated radiation therapy quality assurance and more robust evidence on the relative outcomes, and may help to demonstrate equivalence or otherwise for tumour control and toxicities.

Description of patient populations was also inconsistent within the studies. Seven studies included patient populations comprising both newly diagnosed children receiving first-line therapy as well as those with recurrent disease, but failed to report patient baseline status or outcomes separately [28, 35, 42, 44–46, 48]. We originally planned to include studies with mixed tumour types provided data for individual tumours were reported. Three were identified [58–60] however, after examining these studies we felt that an element of reporting bias could be a factor, as not all the results were consistently reported across the tumour types with the possibility that only exceptional results had been reported, therefore we excluded these studies.

For PBT centres publishing work on expanding cohorts, it is important that it is clear which data has been previously reported, so that the data is not double counted in systematic reviews. Unique cohort identifiers could help this problem [61] such as the system employed for Randomised Controlled Trials [62]. However, this may cause issues with getting studies published as many journals follow the Inglefinger rule, which stipulates that only new previously unpublished data is published [63, 64]. Journals could help by allowing expanding cohorts and encouraging authors to be transparent. This is particularly pertinent to rare disease research where there are fewer patients available to study and where there is a tendency for specific specialist treatment centres to be research active and likely to report on expanding cohorts.

The medical literature has seen a great deal of debate on the necessity or ethical justification of conducting RCTs to evaluate PBT in children. Some commentators contend that equipoise does not apply as the superior dose distributions associated with PBT, must translate into improved patient outcomes and therefore an RCT would not only be unnecessary but unethical [7]. Others argue that it is unethical to use a technology that has had insufficient controlled evaluation of clinically relevant benefit [7, 65]. As well as ethical considerations, differences in the development of radiotherapy treatment compared to drug development also provide challenges in evaluating clinical effectiveness [66, 67]. This may explain why previous paradigm shifts in RT delivery technology, such as IMRT which have been widely implemented, were supported by relatively few RCTs in adults and none in children. The rarity of paediatric CNS tumours, the severity and delayed nature of many of the late effects and willingness of patients and families to undergo randomisation may also render RCTs with late effect endpoints impractical [7, 68] It is, however, recognised that RCTs between PBT and photon therapy are being conducted or planned in adults with cancer including the forthcoming APPROACH trial in adult patients with grade 2 and 3 oligodendroglioma with neurocognitive function as an end point.

This review did not identify any published RCTs, therefore we are unable to answer our primary review questions regarding effectiveness of PBT compared to other radiotherapy treatments in particular photon RT and its role in ameliorating long-term adverse events. Given the increasing use of PBT as standard of care for paediatric brain tumours, perhaps it is too late to ask this question. Indeed, in the UK the large majority of children with primary brain tumours receive radiotherapy with PBT as opposed to photon therapy although this does not apply to many other countries worldwide. We may need to ask how we can maximise the use of PBT both in patients traditionally treated with radiotherapy and patients thus far prohibited such as younger children. If this were the question, again the current body of evidence would have limitations, particularly given the haphazard nature of the research, with few proton centres reporting their activity. Problems with long-term follow-up of patients and little standardisation of the data collected and reported compound the literature. These factors highlighted in this review, stress the need for consistent and systematically collected data on all patients receiving PBT (both trial and non-trial patients) to monitor the effects of treatment including short-term side effects such as radio-necrosis and long term sequelae such as neuro-psychological dysfunction. This is necessary to fully inform clinicians and thus patients and their families of the likely treatment outcome. Indeed such arguments should ideally apply to children receiving photon radiotherapy, and thus may potentially offer a comparison of outcomes between the two techniques albeit in a non-randomised setting. Such comparisons could be subject to future systematic reviews.

Registry data may be one model that could collect data and is a growing area especially with the development of ‘big data’ techniques employed to analyse the data [69]. The success of these ventures is reliant upon the accuracy and consistency of the data input, as well as the continued engagement of stakeholders especially patients, parents, referring teams and of course sufficient long-term funding. Alongside comprehensive prospective databases, there also needs to be a well thought out publications strategy to avoid data duplication/double counting, if separate research teams access one single data source. Although, as discussed above, it is unlikely to see RCTS in children with CNS tumours that will directly compare PBT with photon therapy, RCTs are potentially more feasible with respect to important PBT questions such as delivery techniques (e.g. proton arc therapy), dose and volume, and these are to be encouraged.

In conclusion this review provides a summary of the available data of PBT delivered for a range of CNS tumours arising in children. PBT has been widely implemented in many high-income countries for the treatment of children with cancer including many with CNS tumours. However, in order for the implementation of PBT to continue to evolve, areas where the quality of data could be improved have been highlighted. This may be useful in the context of health systems where cost or geographic access to PBT are issues. Furthermore, improved outcome data, particularly with respect to late effects could inform the continued evolution of the standard indications for PBT.

### Supplementary Information

Below is the link to the electronic supplementary material.

Supplementary file1 (DOCX 23 KB)

Supplementary file2 (DOCX 31 KB)

Supplementary file3 (DOCX 17 KB)

Supplementary file4 (DOCX 36 KB)

Supplementary file5 (DOCX 29 KB)

## Data Availability

Completed data extraction forms are available from the corresponding author on reasonable request. All data included is available in the public domain.

## Abbreviations

3D: Three-dimensional
Adj: Adjuvant
AT/RT: Atypical teratoid/rhabdoid
CFFS: Cystic failure-free survival
CNS: Central nervous system
CSI: Craniospinal irradiation
DFR: Distant failure rate
EFS: Event-free survival
EHF: Extended high-frequency
FSIQ: Full scale Intelligence Quotient
GHD: Growth hormone deficiency
GTR: Gross total resection
Gy_RBE_: SI unit Gray Relative biological effectiveness
HRQoL: Health-related quality of life
IMRT: Intensity-Modulated Radiation therapy
IQ: Intelligence Quotient;
ISRCTN: International Standard Randomised Controlled Trial Number
LFR: Local failure rate
LGG: Low-grade glioma
MGH: Massachusetts General Hospital
NFFS: Nodular failure-free survival
NGGCT: Non-germinomatous germ-cell tumours
NHS: National Health Service
OS: Overall survival
PBT: Proton beam radiotherapy
PFS: Progression-free survival
PNET: Primitive neuroectodermal tumours
PRISMA: Preferred Reporting Items for Systematic Reviews and Meta-analyses
PROSPERO: International prospective register of systematic reviews
RCTs: Randomised controlled trials
RFS: Relapse-free survival
RR: Response rates
RT: Radiotherapy
SIB-R: Scales of independent behaviour revised
SIOP: International Society of Pediatric Oncology
SNHL: Sensorineural hearing loss
STR: Subtotal resection
UCLH: University College London Hospital
UK: United Kingdom
