# Correction to: The effectiveness and safety of proton beam radiation therapy in children and young adults with Central Nervous System (CNS) tumours: a systematic review

**DOI:** 10.1007/s11060-024-04612-7

**Published:** 2024-03-05

**Authors:** Jayne S. Wilson, Caroline Main, Nicky Thorp, Roger E. Taylor, Saimma Majothi, Pamela R. Kearns, Martin English, Madhumita Dandapani, Robert Phillips, Keith Wheatley, Barry Pizer

**Affiliations:** 1 Cancer Research UK Clinical Trials Unit (CRCTU), Institute of Cancer and Genomic Sciences, University of Birmingham, Birmingham, UK; 2 The Clatterbridge Cancer Centre, Liverpool, UK; 3 The Christie Hospital Foundation Trust Proton Beam Therapy Centre, Manchester, UK; 4 College of Medicine, Swansea University, Swansea, UK; 5 Birmingham Women’s and Children’s Hospital NHS Foundation Trust, Birmingham, UK; 6 National Institute for Health Research (NIHR) Birmingham Biomedical Research Centre, University Hospitals Birmingham NHS Foundation Trust, Birmingham, UK; 7 Children’s Brain Tumour Research Centre, University of Nottingham, Nottingham, UK; 8 Queen’s Medical Centre, Nottingham University Hospitals’ NHS Trust, Nottingham, UK; 9 Centre for Reviews and Dissemination (CRD), University of York, York, UK; 10 Alder Hey Children’s NHS Foundation Trust, Liverpool, UK; 11 University of Liverpool, Liverpool, UK

**Correction to: J Neurooncol**

10.1007/s11060-023-04510-4

In this article the reference numbers 60–68 and their citations were incorrect, and number 69 was missing in the References. As a result, publications were cited in the wrong places. The incorrect and correct texts and references are shown under the headings 'INCORRECT…' and 'CORRECT…' below, resp. The affected text is from the middle of the 9th to 12th paragraphs in the **Discussion** section, where reference numbers 60–68 are cited.

The original article has been corrected.

**INCORRECT text and references**

We originally planned to include studies with mixed tumour types provided data for individual tumours were reported. Three were identified [58–60] however, after examining these studies we felt that an element of reporting bias could be a factor, as not all the results were consistently reported across the tumour types with the possibility that only exceptional results had been reported, therefore we excluded these studies.

For PBT centres publishing work on expanding cohorts, it is important that it is clear which data has been previously reported, so that the data is not double counted in systematic reviews. Unique cohort identifiers could help this problem [61] such as the system employed for Randomised Controlled Trials [64]. However, this may cause issues with getting studies published as many journals follow the Inglefinger rule, which stipulates that only new previously unpublished data is published [62, 63]. Journals could help by allowing expanding cohorts and encouraging authors to be transparent. This is particularly pertinent to rare disease research where there are fewer patients available to study and where there is a tendency for specific specialist treatment centres to be research active and likely to report on expanding cohorts.

The medical literature has seen a great deal of debate on the necessity or ethical justification of conducting RCTs to evaluate PBT in children. Some commentators contend that equipoise does not apply as the superior dose distributions associated with PBT, must translate into improved patient outcomes and therefore an RCT would not only be unnecessary but unethical [7]. Others argue that it is unethical to use a technology that has had insufficient controlled evaluation of clinically relevant benefit [7, 65]. As well as ethical considerations, differences in the development of radiotherapy treatment compared to drug development also provide challenges in evaluating clinical effectiveness [66, 67]. This may explain why previous paradigm shifts in RT delivery technology, such as IMRT which have been widely implemented, were supported by relatively few RCTs in adults and none in children. The rarity of paediatric CNS tumours, the severity and delayed nature of many of the late effects and willingnessof patients and families to undergo randomisation may also render RCTs with late effect endpoints impractical [7, 68]

**References**

60. Hwang E, Burnet NG, Crellin AM, Ahern V, Thwaites DI, Gaito S et al. (2022) A novel model and infrastructure for clinical outcomes data collection and their systematic evaluation for UK patients receiving proton beam therapy. Clin Oncol (R Coll Radiol) 34:11–8.62. Toy J (2002) The Ingelfinger Rule. Science Editor 25:195–198
61. Tran S, Lim PS, Bojaxhiu B, Teske C, Baust K, Zepter S et al. (2020) Clinical outcomes and quality of life in children and adolescents with primary brain tumors treated with pencil beam scanning proton therapy. Pediatr Blood Cancer 67:e28465. https://doi.org/10.1002/pbc.28465
62. Asch SM (2018) It’s OK to Talk About It: Exceptions to the Ingelfinger Rule. J Gen Intern Med 33:1825
63. BMC ISRCTN registry. Website—https://www.isrctn.com/. Accessed 26 May 2022
64. Macbeth FR, Williams MV (2008) Proton therapy should be tested in randomized trials. J Clin Oncol 26:2590–2591
65. chnell-Inderst P, Mayer J, Lauterberg J, Hunger T, Arvandi M, Conrads-Frank A et al. (2015) Health technology assessment of medical devices: What is different? An overview of three European projects. Z Evid Fortbild Qual Gesundhwes 109:309–318
66. Miller RC, Lodge M, Murad MH, Jones B (2013) Controversies in clinical trials in proton radiotherapy: the present and the future. Semin Radiat Oncol 23:127–133
67. Luhr A, von Neubeck C, Pawelke J, Seidlitz A, Peitzsch C, Bentzen SM et al. (2018) “Radiobiology of Proton Therapy”: Results of an international expert workshop. Radiother Oncol 128:56–67
68. Major A, Cox SM, Volchenboum SL (2020) Using big data in pediatric oncology: Current applications and future directions. Semin Oncol 47:56–64

**CORRECT text and references**

We originally planned to include studies with mixed tumour types provided data for individual tumours were reported. Three were identified [58-60] however, after examining these studies we felt that an element of reporting bias could be a factor, as not all the results were consistently reported across the tumour types with the possibility that only exceptional results had been reported, therefore we excluded these studies.

For PBT centres publishing work on expanding cohorts, it is important that it is clear which data has been previously reported, so that the data is not double counted in systematic reviews. Unique cohort identifiers could help this problem [61] such as the system employed for Randomised Controlled Trials [62]. However, this may cause issues with getting studies published as many journals follow the Inglefinger rule, which stipulates that only new previously unpublished data is published [63, 64]. Journals could help by allowing expanding cohorts and encouraging authors to be transparent. This is particularly pertinent to rare disease research where there are fewer patients available to study and where there is a tendency for specific specialist treatment centres to be research active and likely to report on expanding cohorts.

The medical literature has seen a great deal of debate on the necessity or ethical justification of conducting RCTs to evaluate PBT in children. Some commentators contend that equipoise does not apply as the superior dose distributions associated with PBT, must translate into improved patient outcomes and therefore an RCT would not only be unnecessary but unethical [7]. Others argue that it is unethical to use a technology that has had insufficient controlled evaluation of clinically relevant benefit [7, 65]. As well as ethical considerations, differences in the development of radiotherapy treatment compared to drug development also provide challenges in evaluating clinical effectiveness [66, 67]. This may explain why previous paradigm shifts in RT delivery technology, such as IMRT which have been widely implemented, were supported by relatively few RCTs in adults and none in children. The rarity of paediatric CNS tumours, the severity and delayed nature of many of the late effects and willingnessof patients and families to undergo randomisation may also render RCTs with late effect endpoints impractical [7, 68].
