# Supplementary material for: The effectiveness and safety of proton beam radiation therapy in children and young adults with Central Nervous System (CNS) tumours: a systematic review
Source: J Neurooncol. 2024 Jan 31;167(1):1–34. doi: 10.1007/s11060-023-04510-4 (PMC10978619; doi:10.1007/s11060-023-04510-4)
Supplement: Supplementary file 1 — Supplementary file1 (DOCX 23 KB) [file 11060_2023_4510_MOESM1_ESM.docx]

**Supplementary information 1 – Search Strategy for EMBASE update**

Database: Embase <1974 to 2021 April 09>

Search Strategy:

--------------------------------------------------------------------------------

1 proton beam radiotherapy.mp. or proton therapy/ (9254)

2 central nervous system tumours.mp. or central nervous system tumor/ (10069)

3 brain tumour.mp. or brain tumor/ (76286)

4 medulloblastoma.mp. or medulloblastoma/ or neuroectoderm tumor/ (22591)

5 ependymoma.mp. or ependymoma/ (10371)

6 atypical teratoid rhabdoid tumor/ or atypical teratoid tumour.mp. (918)

7 ATRT.mp. (738)

8 craniopharyngioma.mp. or craniopharyngioma/ (7790)

9 low grade glioma.mp. or glioma/ (59578)

10 LGG.mp. (3558)

11 germ cell tumour.mp. or germ cell tumor/ (18028)

12 Pineoblastoma.mp. (701)

13 2 or 3 or 4 or 5 or 6 or 7 or 8 or 9 or 10 or 12 (158130)

14 1 and 13 (1135)

15 limit 14 to (human and yr="2019 -Current") (283)
