# Supplementary material for: The effectiveness and safety of proton beam radiation therapy in children and young adults with Central Nervous System (CNS) tumours: a systematic review
Source: J Neurooncol. 2024 Jan 31;167(1):1–34. doi: 10.1007/s11060-023-04510-4 (PMC10978619; doi:10.1007/s11060-023-04510-4)
Supplement: Supplementary file 2 — Supplementary file2 (DOCX 31 KB) [file 11060_2023_4510_MOESM2_ESM.docx]

**Supplementary information 3: Blank data extraction sheet.**

| Study demographics | | |
| --- | --- | --- |
| Author (Year): | |  |
| Investigator group/study name: | |  |
| Hospital and country conducted: | |  |
| Single or multi-institutional study: | |  |
| Study title: | |  |
| Study objectives: | |  |
| Publication status of paper: | |  |
| Is the study linked to other papers? (state): | |  |
| Source of funding and competing interest(s) | |  |
| Study design: | |  |
| Years of study: | |  |
| General tumour type(s): | |  |
| N in overall trial: | |  |
| N receiving PBT / photon RT | |  |
| Sub-tumour types (n): | |  |
| Participants: | | |
| Tumour type(s) (n): | |  |
| Method of diagnosis (biopsy): | |  |
| Age: (median, range: mean; SD): | |  |
| Male: female [%] or male: female (n) | |  |
| Tumour location: | |  |
| Extent of resection: | |  |
| Disease stage: | |  |
| Occurrence of metastasis: | |  |
| Treatment line: | |  |
| Previous treatments (n): | |  |
| Response after previous treatments (n): | |  |
| Inclusion criteria: | |  |
| Exclusion criteria: | |  |
| RT image planning | | |
| CT or MRI image planning; imaging plane and weighting; image contrast enhancement | |  |
| Immobilisation techniques | |  |
| Definition of gross target volume (GTV) and clinical target volume (CTV) tumour volume and margins | |  |
| Intervention(s) | | |
| PBT technique (passive scattering; active scanning; spot scanning; pencil beam; uniform beam scanning) | |  |
| RT type (Gy), fractions, field arrangement/ number of fields | |  |
| Number of administrations | |  |
| Length of time on treatment | |  |
| Concomitant treatments | |  |
| Treatment intent (radical or palliative) | |  |
| Follow-up times | |  |
| Timing of outcome assessments | |  |
| Outcome measures | | |
| Definitions:- | | |
| Overall survival | |  |
| Event-free survival | |  |
| Progression-free survival | |  |
| Disease-free survival | |  |
| Time-to-progression | |  |
| Local control | |  |
| Regional control | |  |
| Short-term adverse events (toxicity) | |  |
| Long-term adverse events | |  |
| Functional status measures | |  |
| Quality of survival | |  |
| Results | | |
| N of patients completing PBT | |  |
| N in analysis | |  |
| Overall survival: | |  |
| Event free survival | |  |
| Progression-free survival | |  |
| Disease-free survival | |  |
| Time-to-progression | |  |
| Local control | |  |
| Regional control | |  |
| Short-term adverse events [toxicity] (n) | |  |
|  | | |
|  | | |
| Quality of survival | |  |
| Comments on results | |  |
| Analysis | | |
| Methods of analysis: | |  |
| Withdrawals and drop-outs: | |  |
| Statistical tests used (state; comment) | |  |
| Conclusions | | |
| Authors conclusions: |  | |
| Reviewers comments: |  | |

| Study Quality Assessment: | | |
| --- | --- | --- |
| 1. | Is the study based on a representative sample selected from the relevant population? |  |
| 2. | Are the recruitment methods adequately described? |  |
| 3. | Is the place of recruitment stated? |  |
| 4. | Are the inclusion/exclusion criteria explicit? |  |
| 5. | Are adequate baseline details on patients reported? |  |
| 6. | Did all patients enter the study at a similar point in their disease progression? |  |
| 7. | Is compliance with treatment adequate? |  |
| 8. | Are any co-interventions stated? |  |
| 9. | Are the outcome measures adequately defined? |  |
| 10. | Are the timings of outcome assessments specified? |  |
| 11. | Were outcomes assessed using objective criteria or was blinding used? |  |
| 12. | Was follow-up long enough for all important events to occur? |  |
| 13. | Are all patients accounted for at the end of the study? |  |
| 14. | Are the statistical analyses appropriate? |  |
| 15. | Generalisability |  |
| 16. | Is there inter-centre variability in the results or is this adequately adjusted for? (if applicable) |  |
| 17. | If comparisons of sub-series are being made, was there sufficient description of the series and distribution of prognostic factors? |  |
| **Reviewers comments (key limitations):** | | |
