# Supplementary material for: The effectiveness and safety of proton beam radiation therapy in children and young adults with Central Nervous System (CNS) tumours: a systematic review
Source: J Neurooncol. 2024 Jan 31;167(1):1–34. doi: 10.1007/s11060-023-04510-4 (PMC10978619; doi:10.1007/s11060-023-04510-4)
Supplement: Supplementary file 3 — Supplementary file3 (DOCX 17 KB) [file 11060_2023_4510_MOESM3_ESM.docx]

**Supplementary information 2 – Data sources**

**Bibliographic databases**

Medical Subject Headings (MeSH) and free-text terms were utilised to build a comprehensive and broad search.

The searches for published studies were undertaken using the following databases: MEDLINE (OvidSP); MEDLINE In-Process Citations and Daily Update (OvidSP); EMBASE (OvidSP); Cochrane Database of Systematic Reviews (CDSR) (Wiley); Cochrane Central Register of Controlled Trials (CENTRAL) (Wiley); CINAHL Plus (EBSCO); PsycINFO (OvidSP); NHS Economic Evaluation Database (NHS EED) (CRD website), DARE (CRD website); and HTA (CRD website). The search strategy used for the MEDLINE search is reported in the Appendix.

**Grey literature**

Completed and ongoing studies were identified by searches of NIH Clinical Trials (http://www.clinicaltrials.gov/); Current Controlled Trials (http://www.controlled-trials.com/); and WHO International Clinical Trials Registry Platform (ICTRP) (http://www.who.int/ictrp/en/).

**Other sources**

Experts in the field, from both the Project Advisory and Patient and Public Involvement (PPI) Groups, were contacted with a list of identified studies to find out whether they had knowledge of any further studies that had not been retrieved by the electronic searches.

Reference lists of all studies included in the present review will be checked, citation searching will be undertaken and the following books of conference abstracts will be hand-searched:

- Annual Meeting of the American Society for Radiation Oncology (ASTRO) (56th and 57th meeting abstracts)
- International Society of Paediatric Oncology (SIOP) (46th and 47th meeting abstracts)
- International Symposium on Pediatric Neuro-Oncology (ISPNO) (15th and 16th meeting abstracts)
- American Society of Clinical Oncology (ASCO) (2014 and 2015)
