# Supplementary material for: The effectiveness and safety of proton beam radiation therapy in children and young adults with Central Nervous System (CNS) tumours: a systematic review
Source: J Neurooncol. 2024 Jan 31;167(1):1–34. doi: 10.1007/s11060-023-04510-4 (PMC10978619; doi:10.1007/s11060-023-04510-4)
Supplement: Supplementary file 4 — Supplementary file4 (DOCX 36 KB) [file 11060_2023_4510_MOESM4_ESM.docx]

**SI Table 1 – Summary of PBT treatment regimens**

| **Study details**  **[Lead author & year (ref)]** | **Tumour type** | **Planned PBT regimen**  **median total dose (range)/fraction dose** | **Treatment volume** | **Technique** |
| --- | --- | --- | --- | --- |
| Eaton (2015)[22]  Shared PBT cohort with Yock (2016)[31] | MB | **PBT** (n=40; 52%):-  CSI: 23.4 Gy_RBE_ (18-27)/1.8 Gy_RBE_  Total IF dose to primary: 54-55.8 Gy_RBE_: 100%  **XRT** (n=37; 48%):-  CSI: 23.4 Gy_RBE_ (18-26.4)/1.8 Gy_RBE_  Total IF dose to primary:  54-55.8 Gy_RBE_: 97%; 55.8 Gy_RBE_: 3% | CSI target volume included the entire subarachnoid volume & nerve roots for all pts.  Location of RT boost:-   - Tumour bed: PBT: 60%; XRT: 51% - PF: PBT: 30%; XRT: PBT: 11% - PF followed by cone-down to tumour bed only:   PBT: 10%; XRT: 6% | PBT delivered as 3D-CPT (100%)  XRT delivered as (3DC) RT (n=13; 35%) or IMRT (n=24; 65%) |
| Eaton (2016)[23]  Shared PBT cohort with Yock 2016[31] | MB | **PBT:-**  CSI: 23.4 (18-27)/1.8 Gy_RBE_   - 18 Gy: n=3 (7%) Gy_RBE_ - 23.4 Gy: n= 41 (91%) - 26.4-27 Gy: n=1 (2%)   IF or PF boost: 54-55.8 :- Gy_RBE_   - Tumour bed (TB): n=28 (62%) - PF: n=13 (29%) - PF > TB: n=4 (9%)   **XRT:-**  CSI: 23.4 Gy (18-26.4)/1.8 Gy   - 18 Gy: n=8 (19%) - 23.4 Gy: 34 (79%) - 26.4-27 Gy: n=1 (2%)   IF or PF boost: 54-55.8 Gy:-   - Tumour bed: n=22 (54%) - PF: n=11 (27%) - PF > TB: n=8 (19) | CSI target volume included the entire subarachnoid volume, nerve roots, & the whole vertebral body in skeletally immature pts (as assessed by age, height, &bone age). | PBT delivered as 3D-CPT  XRT delivered as (3DC) RT or IMRT. |
| Grewal (2019)[24] | MB | CSI: 54 Gy_RBE_/1.8 Gy_RBE_ ns to the tumour bed n=13  50.4 Gy_RBE_, 1.8 /Gy_RBE_ ns n=1 (age <12m) | GTV + 1cm margin where possible with critical structures prioritized. | Double scattered n = 10 (prior to March 2015)  Pencil beam n = 4 (after March 2015) |
| Jimenez  (2013)[25] | MB  /sPNET | CSI: 21.6 Gy_RBE_ (18.0-30.6)/1.8 Gy_RBE_ +  IF boost dose: 54.0 Gy_RBE_ (39.6-54.0): n=11 (73%)  IF only: 52.2 Gy_RBE_ (50.4-54): n=4 (27%) | CSI: CTV = whole-brain field plus length of the spinal canal to the inferior aspect of the thecal sac.  IF: CTV = tumour bed & areas of gross disease + 1.0 cm | 3D-CPT:-  IFRT: 3 or 4-D field conformal plan.  CSI: posterior-anterior spine fields, &both a left &right posterior oblique cranial field |
| Kahalley (2020)[21] | MB | CSI: Standard dose 30.6 to 39.6 or Gy_RBE_ reduced dose 15.0 to 23.4 Gy_RBE_  Total dose to tumour bed ranged from 51 to 59.4GyRBE | CSI: CTV = whole brain &spine.  Tumour bed boost either 1.0cm (SJMB03) or 0.5 cm (SJMB12) CTV margin. | NR |
| Kamran(2018)[26] | MB | NR | NR | NR |
| Moeller (2011)[27] | MB | CSI dose:-  - Standard-risk pts: 23.4 CGE^a,b,c^  - High-risk pts: 36.0 CGE  Boost dose: 54.0-55.8 CGE  Mean cochlear dose: 30.0 CGE (19-43) | NR | NR |
| Paulino (2018)[28] | MB | **PBT** (n=38; 100%):-  CSI: 100%   - standard-risk (n=24; 63%): 18–23.4 Gy_RBE_ /CGE^b,d^ - high-risk (n=14; 37%): 36–39.6 /C Gy_RBE_ GE   Tumour bed boost (54–55.8 Gy_RBE_ /CGE): n=38 (100%)  Cochlear dose:  Mean (± SD): 31.5 Gy_RBE_ /CGE (±7.9)  Median (range): 29.3 Gy_RBE_ /CGE (16.0–52.5)  **XRT (n=46; 100%):-**  CSI: 100%   - standard-risk (n=34; 74%): 18–23.4 Gy^b,d^ - high-risk (n=12; 26%): 36–39.6 Gy   Boost dose:   - Tumour bed boost (54–55.8 Gy): n=11 (24%) - PF boost: n=6 (13%) - PF followed by tumour bed boost (to 36 Gy): n=29 (63%)   Cochlear dose:  Mean (± SD): 37.3 Gy (± 5.4)  Median (range): 35.9 Gy (25.2–54.9) | NR | PBT: Post-2007, pts were treated with passively scattered PBT to the craniospinal axis &the tumour bed at the MD Anderson Proton Center (n = 38).  XRT: Pre-2007, all pts were treated with 3-D photons to the craniospinal axis followed by IMRT to the boost field (n = 46). |
| Sethi (2014)[29] | MB | CSI: 23.4 Gy_RBE_ (18.0-36.0)^a,b^ + either IF boost (n=70; 64%) or whole PF boost (n=39; 36%)^f^ | CTV (brain):- followed conventional anatomical definition of the structure;  CTV (spine) included the thecal sac (dura surrounding the spinal cord, cauda equine, &CSF). | Passive scattered protons. 3-D planned; the brain & spine fields matched using a feathered junction that alternated every day across 3 locations |
| Yock (2014)[30]^g^ | MB /  PNET | NR | NR | NR |
| Yock (2016)[31] | MB | PBT alone: n=53; 90%  PBT + XRT^h^: n=6; 10%  CSI: 23.4 Gy_RBE_ (range; 18-36; IQR: 23.4-27.0)/1.8 Gy  Boost dose: 54.0 Gy_RBE_ (IQR: 54.0-54.0)  XRT: ^d^1.8-10.8 Gy /3.5 Gy | CSI:- skeletally immature children: entire subarachnoid volume, whole vertebral body, &nerve roots; skeletally mature children: subarachnoid volume & spinal nerve roots  Boost dose:-  PF (including brainstem & cerebellum): CTV = GTV + 1–1·5 cm;  Tumour bed: “defined by registering both the preoperative MRI &the postoperative MRI to the planning CTV &contouring the resection cavity &any gross residual disease. This  volume was volumetrically expanded by an additional margin of 1–1·5 cm &anatomically constrained to the PF margin around the resection cavity to encompass any microscopic disease.”  Location of RT boost: tumour bed: n=36 (61%);  PF: n=23 (39%) | Passively scattered PBT. |
| Ares (2016)[32] | Epend | 59.4 Gy_RBE_ (54–60)/1.8-2.0 Gy_RBE_ | CTV = GTV + 0.5–1 cm restricted for anatomical boundaries | Pencil beam scanning proton therapy “using energy-degraded beams from the 590-MeV cyclotron until 2005 & subsequently the dedicated 250-MeV cyclotron. Dose constraints to organs at risk (OARs) were determined as maximum dose of 59.4 Gy_RBE_ to the brainstem, 50.4 Gy_RBE_ to the spinal cord (for pts with infratentorial tumour’s with extension to the upper cervical spine), 54 Gy_RBE_ to the optic chiasm &optic nerves & mean dose to at least one cochlea of 36 Gy_RBE_. |
| Eaton (2015)[33] | Epend | 50.4 Gy_RBE_ (35.0-55.8)/1.8 Gy_RBE_  RT type:-  IFRT: n=14 (70%); HRT: n=1 (5%); HRT/SRS: n=1 (5%)  BT: n=2 (10%); CSI: n=1 (5%); SRS: n=1 (5%) | IF PBT: CTV = GTV + 3-5 mm;  Single fraction or hypo-fractionated proton radiosurgery: CTV = GTV + 0-1 mm;  CSI was prescribed using a modified technique to limit previously irradiated portion of the upper cervical spine & brainstem to 16.2 Gy_RBE_ | 3D-CPT - double scatter proton technique |
| Indelicato (2017)[34] | Epend | All pts: 59.4 Gy_RBE_ (52.2-59.4)^b^  Pts aged ≤ 3 years (n=98: 55%): 54.0 Gy_RBE_ (54.0-59.4)  Photon component: 11 pts due to cyclotron downtime | Treatment given according to University of Florida treatment guidelines for Epend – details given in publication  CTV: GTV + 5mm  modified for anatomic barriers to tumour spread | PBT delivered as sequential, double-scattered protons; three fields delivered daily. A mix of posterior beams &out-of-plane superior oblique’s to avoid multiple beams ending on shared brainstem tissue beyond the CTV. |
| MacDonald (2013)[35] | Epend | 55.8 Gy_RBE_ (50.4-60.0)/1.8 Gy_RBE_ per day  >54Gy 40pts (57%) | CVT = GTV + 0.5-1 cm restricted for anatomical boundaries | Involved Field 3D-CPT |
| Sato (2017)[36] | Epend | PBT: 55.8 Gy_RBE_ (50.4-59.4)/1.8 Gy_RBE_ (5 days/wk)  XRT: 54.0 Gy (50.0-59.0)/1.8 Gy (5 days/wk) | PBT: CTV = tumour bed & any residual tumour + 0.5-1-cm margin;  XRT: CTV = tumour bed & any residual tumour + 1.0 cm margin | XRT was delivered as IMRT |
| De Amorim Bernstein (2013)[37] | AT/RT | IF (n=7; 70%): 50.4 Gy_RBE_ (50.4-55.8)/1.8 Gy_RBE_  CSI (n=3; 30%): 23.4 Gy_RBE_ (18.0-23.4)/1.8 Gy_RBE_ (age >3yrs)  Tumour boost dose: 55.8 Gy_RBE_ (54-55.8)^i^ | CTV = GTV + 0.5cm | 3D-CPT |
| Haskins (2015)[38] | AT/RT | Local (n=9; 56%): 54.0 Gy_RBE_ (48.6-59.4)/1.8 Gy_RBE_  CSI (n=7; 44%): as above + 30.6 Gy_RBE_ (23.4-36.0)/1.8 Gy_RBE_ | CTV = GTV + 0.5-1.0cm | 3D-CPT via uniform active scanning |
| McGovern (2014)[39] | AT/RT | Local (n=17; 55%):-  50.4 Gy_RBE_ (9.0-54.0)/1.8 Gy_RBE_  CSI (n=14; 45%):-   - - ≤ 24 Gy_RBE_ or less : n=7 (22.5%) - - ≥ 30.6 Gy_RBE_ or more n=7 (22.5%)   + tumour dose: 54.0 Gy_RBE_ (43.2-55.8)/1.8 GY_RBE_^j^ | CVT = GTV + 1cm | Passive scatter proton therapy. For pts receiving CSI, the brain treated with opposed oblique fields & spine with posterior-anterior spinal fields. |
| Weber (2015)[6] | AT/RT | Focal (n=15; 100%): 54.0 Gy_RBE_/1.8 Gy_RBE_ | CTV = GTV + 1cm | Pencil beam scanning proton therapy.  Dose constraints to OAR determined as max dose of 50 & 54 Gy_RBE_ to centre & surface of the brainstem or spinal cord, 50 Gy_RBE_ to the optic chiasm, 45 Gy_RBE_ to the optic nerves, mean/max of 20/30 Gy_RBE_ to the lacrimal glands, mean/max dose of 36/45 Gy_RBE_ to the cochlea’s & mean/max dose of 7/10 Gy_RBE_ |
| Bass (2018)[41] | Cranio | 54 Gy_RBE_ /1.8 Gy_RBE_ | CTV = GTV + margins for microscopic diseases & setup uncertainty. | NR |
| Bishop (2014)[42] | Cranio | PBT: 50.4 Gy_RBE_ (50.4-54)/1.8 Gy_RBE_  XRT: 50.4 Gy (50.4-54)/1.8 Gy | **-** | PBT: mostly delivered by passive scattered techniques (86%).  XRT was delivered as IMRT. |
| Jimenez (2021)[43] | Cranio | 52.2 Gy_RBE_ (45-54) /1.8 Gy_RBE_ | NR | Passively scattered 76pts, pencil beam 1pt. Conformal PBT delivered using 3 to 4 field. |
| Laffond (2012)[44]^k^ | Cranio | 54.0 Gy_RBE_ (54.0-55.2)^b^ | NR | First nine pts treated with a combination of PBT & XRT; thereafter, PBT used exclusively for the remainder of pts. |
| Luu (2006)[45] | Cranio | 50.4-59.4 CGE/1.8 CGE^d^ | NR | 3D-CPT delivered daily. “Beam arrangements consisted mainly of right lateral, left lateral & vertex fields. Beam directions were chosen to provide sufficient coverage of the target volume with a 3 mm margin for uncertainty while limiting the dose to critical surrounding normal structures to within the tolerance of those structures”. |
| Winkfield (2009)[46] | Cranio | 52.2 Gy_RBE_ (range: 52.2-54.0)/1.8 Gy_RBE_ | CTV = GTV + 1cm | Conformal PBT, typically delivered using a four-field approach using laterals, one anterosuperior field & one posterior field. |
| Greenberger (2014)[47] | LGG | PBT alone: n=23 (72%)  PBT plus XRT: n=9 (28%)  52.2 Gy_RBE_ (48.6-54.0)/1.8 Gy_RBE_ | CTV = GTV (defined as resection cavity & any gross tumour visible on MRI/CT) + 3-5 mm expansion around the GTV. | Supratentorial lesions: 4-field plan (2 lateral, 1 x superior & 1 x posterior field); Infratentorial lesions: 3-field plan (2 x posterior oblique & 1 x posterior field);  Spinal treatment: 1-3 posterior or oblique posterior fields. An additional margin of 8mm-12mm was added account for set-up uncertainty & penumbra of the proton beam. |
| Hug (2002)[48] | LGG | 55.2^a^ CGE (50.4-63)/1.8 CGE per day, five per week | CTV = GTV + 0.5–1 cm | 3D-CPT. GTV included any enhancement. Critically normal tissues were delineated including the optic nerves, optic chasm, brainstem, pituitary gland & cochlea. |
| Indelicato (2019)[49]  UFCM  2007-2017 | LGG | All pts:  54 : Gy_RBE_ n=129 (74%); < 54 : Gy_RBE_ n=45 (26%)  Pts aged ≤ 5 yrs:  54 : n Gy_RBE_ =17 (10%); < 54 Gy_RBE_ : n=9 (5%) | CTV = GTV + 0.5cm with further modification as necessary to encompass all surfaces originally in contact with the tumour. The expansion used for the planning target volume (PTV) was a uniform 3 mm, applying daily image guidance. | PBT delivered via double-scattered protons. |
| MacDonald (2011)[50] | GCT | **Germinoma (n=13; 59%):-**  CSI (n=5; 38%): 20.4 Gy_RBE_ (18.3-23.4) with IF boost: 14.4 Gy_RBE_ (7.2-36.0)_;_ total dose: 36.3 Gy_RBE_ (30.6-57.6)^b^  WVRT (n=7; 54%): 21.0 Gy_RBE_ (19.5-23.4) with IF boost: 16.2 Gy_RBE_ (7.2-22.0)_;_ total dose: 37.5 Gy_RBE_ (30.6-45.4)  WBRT (n=1; 5%): 25.5 Gy_RBE_ with IF boost: 19.8 Gy_RBE_;  total dose: 45.3 Gy_RBE_  **NGGCT (n=9; 41%):-**  CSI (89%): 25.2 Gy_RBE_ (21.6-36.0) with IF boost: 24.3 Gy_RBE_ (18-30.6); Total dose: 52.2 Gy_RBE_ (45.0-57.6)^b^  IF boost (n=1; 11%): 50.4 Gy_RBE_ | NR | 3D-CPT |
| Farnia (2014)[51]^l^ | Pineo | CSI dose = 36 Gy_RBE_ (range 23.4 – 40Gy) Median focal dose 54 Gy_RBE_ (range 40 – 58.4 Gy_RBE_) 24 pts reporting, both photon & PBT, fractions not reported. | NR | NR |

**Key**:

**a:** mean; **b:** fractions not reported; **c:** range not reported; **d:** median dose not reported, range only provided; **e:** dose information relates to MB/PNET patients only (n=12), rather than the total number of patients in the study (n=22); **f:** boost dose not reported; **g:** data not reported separately for MB/PNET patients, the only tumour type which satisfied the minimum patient numbers necessary for inclusion in this review; **h:** Six patients were treated with photon RT for a portion of their treatment because of cyclotron breakdown (dose: 1.8-10.8 Gy). These patients were treated in the analysis on an intention-to-treat basis; **i:** Patients over 3 years received CSI with a boost dose to the tumour bed; **j:** 1 patient received CSI at 1.6 Gy_RBE_ in line with the European Rhabdoid Registry (EU-RHAB) protocol; **k:** PBT was combined with photon RT in the first nine patients, subsequent patients received only PBT; **l:** This study consisted of both adults and children who underwent PBT or photon RT. However, this information relates solely to children who underwent PBT.

**(3DC) RT: (three-dimensional conformal) radiotherapy; 3D-CPT: three-dimensional conformal proton therapy; Adju: adjuvant; AT/RT: Atypical Teratoid/Rhabdoid Tumour; BT: iodine-131 brachytherapy; CGE: cobalt gray equivalent; cGy: centigray; CSI: Craniospinal irradiation; Cranio: Craniopharyngioma; CT: computed tomography imaging; CTV: Clinical target volume; Diag: diagnosis; Epend: Ependymoma; Gy: gray; GCT: Germ Cell Tumour; GTV: Gross tumour volume; IF: Involved Field; IFRT: Involved field radiation therapy; HRT: hypofractionated RT; IMRT: Intensity-modulated radiation therapy; INF: Infratentorial; LGG: Low grade glioma; MB: Medulloblastoma; NR: not reported; PF: Posterior Fossa; PIN: Pineoblastoma; PNET: supratentorial primitive neuroectodermal tumours; RBE: relative biological effectiveness; RT: radiotherapy; SD: standard deviation; SRS: stereotactic radiosurgery; SUP: Supratentorial; WBRT: whole brain radiotherapy; WVRT: whole ventricular radiotherapy**
