# Supplementary material for: The effectiveness and safety of proton beam radiation therapy in children and young adults with Central Nervous System (CNS) tumours: a systematic review
Source: J Neurooncol. 2024 Jan 31;167(1):1–34. doi: 10.1007/s11060-023-04510-4 (PMC10978619; doi:10.1007/s11060-023-04510-4)
Supplement: Supplementary file 5 — Supplementary file5 (DOCX 29 KB) [file 11060_2023_4510_MOESM5_ESM.docx]

**SI Table 2. Health Related Quality of Life (HRQoL) Results**

| **Study details** | **Tumour type** | **N in study**  **(assessed)** | **Median follow-up (range)** | **HRQoL**  **Measurement scale employed** | **Results** |
| --- | --- | --- | --- | --- | --- |
| Grewal (2019)[24] | MB | 14  (9) | Median: 4 yrs (1 – 7yrs) | Lansky & School PS | “Although some pts had an initial decline in Lansky performance status, these scores returned to baseline within two years after tumor bed irradiation, as seen in Figure 3. One patient had a decline in Lansky performance status from 100 to 90 at year 3, with a return to 100 by year 4. All children returned to school after treatment completion, with four requiring an individualized education plan for: hearing loss (1), ADHD(1), mixed receptive-expressive language disorder in addition to ADHD (1), & autism spectrum disorder”. |
| Kamran (2018)[26] | MB / PNET | 116  (63-110) | Median:  5 yrs  (1-10.6)  Mean:  5.03 yrs | PedsQL | **Mean PedsQL scores at baseline & annual change in mean PedsQL scores [p-value] – total & by sub-domain**  **Total core score: Child Report (n=81):-**  **Baseline: 65.9 (95% CI: 62.3-69.4); Annual change: 1.8 points (95% CI: 1.2-2.4) [p<.001]**  **Total core score: Parent-Proxy report (n=107):-**  **Baseline: 59.1 (95% CI: 55.6-62.3); Annual change: +2.0 points (95% CI: 1.4-2.7) [p<0.001]**  *Physical Score: Child Report (n=85)*  Baseline: 58.2 (95% CI: 53.1-63.4); Annual change: +3.3 (95% CI: 2.6-4.1) [p<0.001]  *Physical Score: Parent-Proxy Report (n=108)*  Baseline: 49.9 (95% CI: 44.8-54.9); Annual change: +4.0 (95% CI: 3.1-4.8) [P< 0.001]  *Psychosocial Score: Child Report (n=81)*  Baseline: 70.8 (95% CI: 67.4-74.2); Annual change: +0.9 (95% CI: 0.2-1.5) [p=0.006]  *Psychosocial Score: Parent-Proxy Report (n=109)*  Baseline, 65.8 (95% CI: 62.8-68.9); Annual change: +0.8 (95% CI: 0.2-1.4) [p=0.007]  Psychosocial sub-scores:-  *School Score: Child Report (n=63)*  Baseline: 56.4 (95% CI: 50.1-42.1); Annual change: +1.8 (95% CI: 0.0-2.7) [p<0.001]  *School Score: Parent-Proxy Report (n=69)*  Baseline: 49.3 (95% CI: 42.1-56.4); Annual change: +2.0 (95% CI: 1.1-2.9) [p<0.001]  *Social Score: Child Report (n=85)*  Baseline: 80.0 (95% CI: 76.1-83.9); Annual change: +0.6 (-0.1 to 1.2) [p=0.100]  *Social Score: Parent-Proxy Report (n=110)*  Baseline: 76.0 (95% CI: 73.0-79.0); Annual change: -0.4 (95% CI: -1.1 to 0.3) [p=0.270]  *Emotional Score: Child Report (n=85)*  Baseline: 73.0 (95% CI: 69.0-77.0); Annual change: +0.7 (95% CI: 0.04-1.4) [p=0.064]  *Emotional Score: Parent-Proxy Report (n=109)*  Baseline: 63.7 (95% CI: 60.3-67.0); Annual change: +1.4 (95% CI: 0.8-2.1) [p<0.001]  **Authors’ conclusions: “HRQoL scores appear to increase over time after treatment in children treated with proton RT for MB but remain lower compared with those of parent-proxy reports as well** **as published means from a healthy normative sample of children.”** |
| Yock  (2014)[30] | MB | 48 (47) | Median for MB only:-  PBT: 3.0 yrs  IMRT:  2.4 yrs | PedsQL | **Mean (SD) PedsQL scores for MB/PNET only: PBT vs XRT**  **Total PedsQL core score:-**  **PBT (n=19): 76.3 (14.0); XRT (n=28): 66.5 (17.7); p=0.05**  *Physical summary score: -*  PBT (n=19): 81.1 (18.6); XRT (n=28): 68.1 (22.5); **p=0.044**  *Psychosocial summary score:-*  PBT (n=19): 73.8 (14.3); XRT (n=28): 66.0 (17.5); p=0.113  Psychosocial sub-scores:-  *School functioning:-*  *PBT (n=19): 66.3 (25.5); XRT (n=23): 67.8 (19.2); p=0.828*  *Social functioning:-*  *PBT (n=19): 79.0 (13.8); XRT (n=28): 62.3 (23.1); p=0.003*  *Emotional functioning:-*  *PBT (n=19): 76.1 (15.3); XRT (n=29): 69.1 (20.2); p=0.211*  **Authors’ conclusions: “The HRQoL of pediatric brain tumor survivors treated with PBT compare favorably to those treated with XRT”.** |
| Weber (2015)[40] | AT/RT | 15 (4-9) | 2.8 yrs  (0.8 – 5.8) | PedsQoL – parental proxy version | Parental proxy mean PedsQoL scores [±SD] at baseline (pre-PBT) & follow-up (two-months post-completion of PBT) – total & by sub-domain:  **Total Mean Score [±SD]:-**  **Baseline (n=8): 44.20 [±18.53]; Follow-up (n=7): 42.01 [±17.84]**  *Physical Mean Score:-*  Baseline (n=8): 39.59 [±22.31]; Follow-up (n=8): 43.59 [±21.03]  *Emotion Mean Score:-*  Baseline (n=9): 41.53 [±18.98]; Follow-up (n=8): 44.19 [±21.04]  *Social Mean Score:-*  Baseline (n=7): 47.07 [±28.44]; Follow-up (n=7): 35.86 [±26.79]  *Kindergarten/School Mean Score:-*  Baseline (n=4): 56.25 [±4.17]; Follow-up (n=4): 62.50 [±8.33]  *Psycho-social Mean Score:-*  Baseline (n=7): 45.35 [±16.91]; Follow-up (n=7): 43.71 [±15.43]  **Authors’ conclusion: “Our prospective parental-proxy reporting data do not** **suggest a decrease of QoL of these very young pts.”** |
| Laffond (2012)[44] | Cranio | 29 (22 for both self- & parent-proxy reports) | Median follow-up: 6.17 yrs (SD: 4.5) | HRQoL: Kidscreen-52  (self- & parental-proxy questionnaires);  Mood disorders:  MDI-C ;  Executive Function: (BRIEF) | Mean T-score (SD) for each Kidscreen 52 dimension, self-report & proxy-report (% of pts with a T-score <40*). **[p-value where self- & parent proxy-report significantly differed]**  *****NB: T-scores below 40 (± 1 SD) considered indicative of low HRQoL (mean=50; SD=10).    **Kidscreen 52 dimensions:**  *Physical well-being:*  Self-report: 43.05 (10.01); 36.4%; Proxy-report: 39.23 (9.7); 62% [**p=0.04**]  *Psychological well-being:*  Self-report: 47.64 (11.0); 22.7%; Proxy-report: 43.9 (13.3); 50%  *Moods & emotions:*  Self-report: 51.14 (13.2); 27.3%; Proxy-report: 51.2 (13.5); 13.6%  *Autonomy:*  Self-report: 45.68 (10.7); 27.3%; Proxy-report: 43.77 (8.2); 32%  *Self-perception:*  Self-report: 48.31 (10.35); 27.3%; Proxy-report: 44.04 (11.9): 41%  *Parents relations/homelife:*  Self-report: 45.04 (9.8); 27.3%; Proxy-report: 48.68 (13.6); 36.4%  *Financial resources:*  Self-report: 47.57 (12.3); 23,8%; Proxy-report: 47.0 (10); 19%  *Social support & peers:*  Self-report: 43.36 (14.5); 50%; Proxy-report: 36.19 (13.0); 16% [**p=0.02**]  *School environment:*  Self-report: 49.63 (10.01); 13.6%; Proxy-report: 48.09 (11.4); 22.7%  *Social acceptance:*  Self-report: 44.63 (13.1); 32%; Proxy-report: 45.54 (10.7); 31.8%  **MOOD DISORDERS**  **Mean MDI-C T-score for the group: 49.1 (SD=13.3)**  Depressive symptoms (T-score > 55): n=11:-   - slight-to-moderate (T-score 56-65): n=8 - moderate-to-severe (T-score 66-75): n=3 - Severe (T-score >75): n=0 - Referral to a psychiatrist for suicidal ideas: n=1   **EXECUTIVE FUNCTIONING**  Brief sub-scales – Mean T-score (SD); number (%) of pts with a T-score ≥ 65:  *Behavioural Regulation index - 53.4 (11.3); 5 (24)*   - Inhibit - 44.7 (10.8); 0 - Shift - 56.6 (13.5); 8 (38) - Emotional control - 57.8 (14.8); 8 (38)   *Metacognition index – 54.3 (16.2); 6 (30)*   - Initiate - 53.2 (12.3); 6 (29) - Working memory - 52.9 (15.2); 6 (29) - Plan/organise - 52.9 (15.2); 5 (24) - Organization of materials (n=20): 50.2 (10.5); 1 (5) - Monitor - 49.8 (10.9); 3 (14)   **Global Executive Composite Score: 52.2 (12.9); 6 (30)**  Overall, 24–38% of the parents reported elevated scores in the BRIEF sub-scales & problems were particularly noted for ‘shift’ & emotional control’ in the behavioural domain & for ‘initiation’, ‘working memory’ & ‘organization’ in the metacognitive domain. Overall, the GEC fell in the clinical range for one third of the group.  **Author’s** **conclusions: “No significant change in either self-report or parental** **proxy scores from baseline at 3.4 years follow-up. Half of the group had mild-to-moderate depressive symptoms in the MDI-C questionnaire. Executive disorders in everyday life were highlighted by the BRIEF questionnaire in a third of the group. Finally, low HRQoL, depressive & dysexecutive symptoms were significantly correlated to each other.”** |
| Hug  (2002)[48] | LGG | 27 (27) | 3.3 yrs  (0.6-6.8) | Lanksy Performance Scale | According to authors, ‘No patient experienced a drop of more than 10% on the Lansky performance scale.’ |

Key: **AT/RT: Atypical Teratoid/Rhabdoid Tumour; Cranio: Craniopharyngioma; HRQoL: health-related quality of life; LGG: Low Grade Glioma; MB: Medulloblastoma; PBT: Proton Beam Therapy; Photon RT: Photon Radiotherapy; PNET: Primitive Neuroectodermal Tumour; pts: patients**
